# Computationally prioritized drugs inhibit SARS-CoV-2 infection and syncytia formation

**DOI:** 10.1093/bib/bbab507

**Published:** 2021-12-27

**Authors:** Angela Serra, Michele Fratello, Antonio Federico, Ravi Ojha, Riccardo Provenzani, Ervin Tasnadi, Luca Cattelani, Giusy del Giudice, Pia A S Kinaret, Laura A Saarimäki, Alisa Pavel, Suvi Kuivanen, Vincenzo Cerullo, Olli Vapalahti, Peter Horvath, Antonio Di Lieto, Jari Yli-Kauhaluoma, Giuseppe Balistreri, Dario Greco

**Affiliations:** Faculty of Medicine and Health Technology, Tampere University, Tampere, Finland; BioMediTech Institute, Tampere University, Tampere, Finland; Finnish Hub for Development and Validation of Integrated Approaches (FHAIVE), Tampere, Finland; Faculty of Medicine and Health Technology, Tampere University, Tampere, Finland; BioMediTech Institute, Tampere University, Tampere, Finland; Finnish Hub for Development and Validation of Integrated Approaches (FHAIVE), Tampere, Finland; Faculty of Medicine and Health Technology, Tampere University, Tampere, Finland; BioMediTech Institute, Tampere University, Tampere, Finland; Finnish Hub for Development and Validation of Integrated Approaches (FHAIVE), Tampere, Finland; Department of Virology, Faculty of Medicine, University of Helsinki, Helsinki, Finland; Drug Research Program, Faculty of Pharmacy, University of Helsinki, Helsinki, Finland; Synthetic and Systems Biology Unit, Biological Research Centre, Eotvos Lorand Research Network, Szeged, Hungary; Faculty of Medicine and Health Technology, Tampere University, Tampere, Finland; BioMediTech Institute, Tampere University, Tampere, Finland; Finnish Hub for Development and Validation of Integrated Approaches (FHAIVE), Tampere, Finland; Faculty of Medicine and Health Technology, Tampere University, Tampere, Finland; BioMediTech Institute, Tampere University, Tampere, Finland; Finnish Hub for Development and Validation of Integrated Approaches (FHAIVE), Tampere, Finland; Faculty of Medicine and Health Technology, Tampere University, Tampere, Finland; BioMediTech Institute, Tampere University, Tampere, Finland; Finnish Hub for Development and Validation of Integrated Approaches (FHAIVE), Tampere, Finland; Institute of Biotechnology, University of Helsinki, Helsinki, Finland; Faculty of Medicine and Health Technology, Tampere University, Tampere, Finland; BioMediTech Institute, Tampere University, Tampere, Finland; Finnish Hub for Development and Validation of Integrated Approaches (FHAIVE), Tampere, Finland; Faculty of Medicine and Health Technology, Tampere University, Tampere, Finland; BioMediTech Institute, Tampere University, Tampere, Finland; Finnish Hub for Development and Validation of Integrated Approaches (FHAIVE), Tampere, Finland; Department of Virology, Faculty of Medicine, University of Helsinki, Helsinki, Finland; Drug Research Program, Faculty of Pharmacy, University of Helsinki, Helsinki, Finland; Department of Virology, Faculty of Medicine, University of Helsinki, Helsinki, Finland; Department of Veterinary Biosciences, University of Helsinki, Helsinki, Finland; Department of Virology, University of Helsinki and Helsinki University Hospital, Helsinki, Finland; Institute for Molecular Medicine Finland, University of Helsinki, Helsinki, Finland; Synthetic and Systems Biology Unit, Biological Research Centre, Eotvos Lorand Research Network, Szeged, Hungary; Department of Forensic Psychiatry, Aarhus University, Aarhus, Denmark; Drug Research Program, Faculty of Pharmacy, University of Helsinki, Helsinki, Finland; Department of Virology, Faculty of Medicine, University of Helsinki, Helsinki, Finland; Queensland Brain Institute, The University of Queensland, Brisbane, Australia; Faculty of Medicine and Health Technology, Tampere University, Tampere, Finland; BioMediTech Institute, Tampere University, Tampere, Finland; Finnish Hub for Development and Validation of Integrated Approaches (FHAIVE), Tampere, Finland; Institute of Biotechnology, University of Helsinki, Helsinki, Finland

**Keywords:** COVID-19, SARS-CoV-2, drug repositioning, drug design, virtual screening, 7-hydroxystaurosporine, bafetinib, syncytia, kinase inhibitors, delta variant

## Abstract

The pharmacological arsenal against the COVID-19 pandemic is largely based on generic anti-inflammatory strategies or poorly scalable solutions. Moreover, as the ongoing vaccination campaign is rolling slower than wished, affordable and effective therapeutics are needed. To this end, there is increasing attention toward computational methods for drug repositioning and *de novo* drug design.

Here, multiple data-driven computational approaches are systematically integrated to perform a virtual screening and prioritize candidate drugs for the treatment of COVID-19. From the list of prioritized drugs, a subset of representative candidates to test in human cells is selected. Two compounds, 7-hydroxystaurosporine and bafetinib, show synergistic antiviral effects *in vitro* and strongly inhibit viral-induced syncytia formation. Moreover, since existing drug repositioning methods provide limited usable information for *de novo* drug design, the relevant chemical substructures of the identified drugs are extracted to provide a chemical vocabulary that may help to design new effective drugs.

## Introduction

The rapid diffusion of the COVID-19 pandemic has called for a prompt reaction from the biomedical research community. Although new vaccines have been developed as preventive options against the infection spreading [1, 2], the ongoing vaccination campaign is still rolling significantly slowly in many areas of the planet. Furthermore, even if monoclonal antibody-based therapies represent an appealing option to treat the most severe cases of COVID-19, they are expensive and not easy to mass produce [3]. Thus, more effective and affordable treatments for COVID-19 are still required to support medical intervention for the disease worldwide.

The SARS-CoV-2 entry in the cells is mediated by the virus spike protein that binds the angiotensin I converting enzyme 2 (ACE2) receptor of the host [4]. The spike protein initiates the viral–cell membrane fusion, thus delivering the viral RNA in the host cell cytoplasm. The spike protein reaction is dependent on proteolytic cleavage, as well as activation of viral envelope glycoproteins, by host cell proteases, such as transmembrane protease, serine 2 (TMPRSS2), cathepsin L (CTSL) and cathepsin B (CTSB) [5]. SARS-CoV-2 can use both the endosomal cysteine (CTSB/CTSL) and serine (TMPRSS2) proteases to prime the host cells, since the full inhibition of viral entry can only be attained by the presence of both protease inhibitors [6]. Currently available therapeutic options target stages of the viral life cycle (e.g. nucleotide analogs or broad-spectrum antiviral drugs), the host immunological response (anti-inflammatory drugs or monoclonal antibodies) or vascular acute damage (antihypertensive and anticoagulant drugs) [7–12]. ACE2 receptor and TMPRSS2 may serve as therapeutic targets due to their crucial role in the initial phases of the viral infection [6, 13].

Antivirals, especially antiretrovirals, represent the class of therapeutic agents that has been investigated to a larger extent [14, 15]. Various other drug classes were also proposed, such as anticancer drugs (e.g. kinase inhibitors) and antimicrobials [16, 17]. The majority of clinical trials focuses on hydroxychloroquine alone or in combination with other compounds (38.65%), followed by immunotherapeutic (33.13%) and antiviral agents (9.20%) [18].

Given the cost and time required for *de novo* drug development, drug repositioning is emerging as a viable solution [19–22]. Several efforts have been made to experimentally screen large libraries of candidate compounds [23–26]. Traditional screening e.g. high-throughput screening (HTS), represents the first step in modern drug development, testing thousands to millions of small molecules in parallel. HTS ‘hits’ allow the identification of therapeutic targets and to validate biological effects even when little is known about the compound. However, HTS has a substantial cost in terms of time and resources, requiring the experimental testing of libraries of hundreds of thousands of small molecules to obtain a few active compounds for further investigation. This translates into typical hit rates between 0.01% and 0.14% [27]. In contrast, virtual screening can improve the success rate and reduce costs in the early phases of drug development. Virtual libraries are not constrained to logistic aspects (e.g. availability, cost, storage), allowing to computationally assay heterogeneous libraries of small molecules against the desired targets, resulting in a reduced set of candidate molecules (few tens). In addition, drug-likeness or absorption, distribution, metabolism, excretion and toxicity (ADMET) criteria can be embedded into the process to further increase the quality of the selected candidates [28]. Although virtual screening results still need to be experimentally validated, the number of experiments can be limited to a handful of molecules.

Several computational strategies to drug repositioning for COVID-19 have been proposed. For example, Le *et al*. and Mousavi *et al*. identified candidate therapies based on the analysis of omics data to characterize both the mechanism of action (MOA) of drugs as well as the molecular alterations of the COVID-19 disease [29, 30]. Complementary bioinformatic approaches based on network analysis have also recently been employed to identify potential drugs for COVID-19 treatment [12, 31, 32].

Gordon *et al*. [26] used a computational approach to identify human proteins with high affinity for structural SARS-CoV-2 components. Subsequently, they selected drugs known to target these proteins and performed *in vitro* screening.

Cheminformatics strategies, based on quantitative structure–activity relationship (QSAR) modeling and molecular docking, have also been developed. For example, Alves *et al*. developed QSAR models to predict the compound inhibitory activity of the main protease (Mpro) of the SARS-CoV-2 and employed these models to perform a virtual screening of the DrugBank database [33]. On the other hand, Amin *et al*. exploited previous knowledge about the SARS-CoV virus and performed a QSAR screening to identify an active set of SARS-CoV papain-like protease (PLpro) inhibitors, which were then used to virtually screen an in-house chemical library and was further validated by a molecular docking analysis on a homolog model of the SARS-CoV-2 PLpro [33].

Both bioinformatic and chemoinformatic approaches have their strengths and limitations. Although cheminformatic strategies for drug repositioning usually include a step of virtual screening, bioinformatic approaches are limited to the study of existing drugs and offer little or no viable information to be used in the context of *de novo* drug development. On the other hand, cheminformatic approaches often neglect the biological MOA as well as the downstream biological effects of the drugs. Comprehensive integrated approaches, bridging bioinformatics and cheminformatics, are still missing. Hence, we propose a novel integrated computational approach to prioritize candidate drugs for treating COVID-19. We prioritized compounds from the whole DrugBank library by virtual screening and selected 23 candidates for *in vitro* infection assays to reveal drugs with potential antiviral activity against SARS-CoV-2. 7-Hydroxystaurosporine and bafetinib showed significant antiviral effect in human epithelial cells. Interestingly, the two drugs also revealed a synergistic effect in blocking virus-induced syncytia formation. The strength of our strategy is the integration of cheminformatic and bioinformatic methodologies. Our analytical framework allows the prioritization of a few drugs based on the estimated associations between chemical substructures and desired molecular effects, as potential therapies for COVID-19. Moreover, the set of relevant chemical substructures could also serve as a molecular fragment library for *de novo* drug design or lead optimization.

All the scripts, data and images used in this work are available at https://doi.org/10.5281/zenodo.5643558.

**Figure 1:**
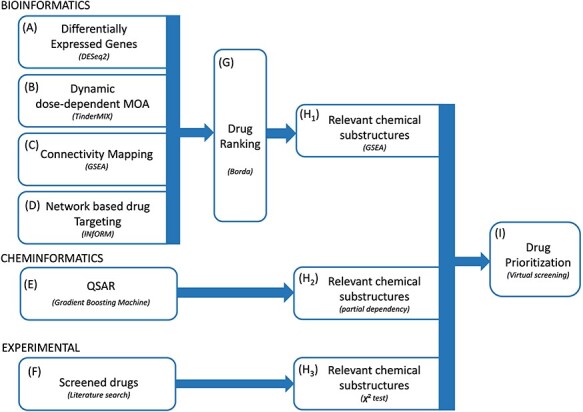
Proposed methodology. We integrated multiple bioinformatics and cheminformatics methods to prioritize drugs for the treatment of COVID-19 (**A**–**E**). Our framework consists of four complementary bioinformatics approaches, including differential expression analysis (**A**), dynamic dose-dependent MOA (**B**), connectivity mapping (**C**) and network-based drug targeting (**D**) as well as a QSAR-based cheminformatics method (**E**). We further complemented our set of candidate chemical substructures with those extracted from active drugs as experimentally tested in multiple studies (**F**). The four bioinformatics approaches are merged to find a robust rank of the drugs (**G**). From the rank produced by the bioinformatic approaches, the QSAR method and from the list of screened drugs, three lists of chemical substructures are identified (**H**_**1**_–**H**_**3**_) with the aim of increasing the robustness of the predictions as well as to generate knowledge readily usable in the context of *de novo* drug development. Eventually, we exploited the set of candidate chemical substructures by performing a virtual screening analysis of the DrugBank database (**I**).

## Results and discussion

### An integrated computational methodology for drugs prioritization

We integrated multiple bioinformatics and cheminformatics methods to prioritize drugs for the treatment of COVID-19 (Figure 1). Our framework is designed to work with four complementary bioinformatics approaches (Figure 1**A**–**D**). We compared the molecular MOA, defined as the set of all the differentially expressed genes (DEGs), of the COVID-19 and of the drugs under study (Figure 1**A**). Since intricate regulatory patterns of molecular regulation are activated to achieve adaptation to a drug exposure, particular emphasis is given to the molecular alterations that follow a dynamic dose-dependent pattern. Thus, we further investigated the MOA of the drugs to identify its portion with a clear point-of-departure (POD), which is denoted as the specific dose-time point at which a given molecule is altered from the steady state in a monotonic manner (Figure 1**B**). Under the hypothesis that an effective drug should be able to counterbalance the perturbation caused by a disease, we used a connectivity mapping [34] based method to identify effective drugs capable of reverting the alteration induced by the disease (Figure 1**C**). Recently, several drug repositioning approaches have been developed, based on the analysis of gene co-expression networks and drug–targets relationships [35, 36]. The main assumption behind these approaches is that genes that are topologically central in the network have a pivotal role in the adaptation to exposure. Consequently, we prioritized the drugs according to the importance of their gene targets in the network (Figure 1**D**). By merging these approaches, we identified a robust rank of the drug by means of the Borda method (Figure 1**G**) and we extracted from there a list of relevant chemical substructures (Figure 1**H**_1_).

**Figure 2:**
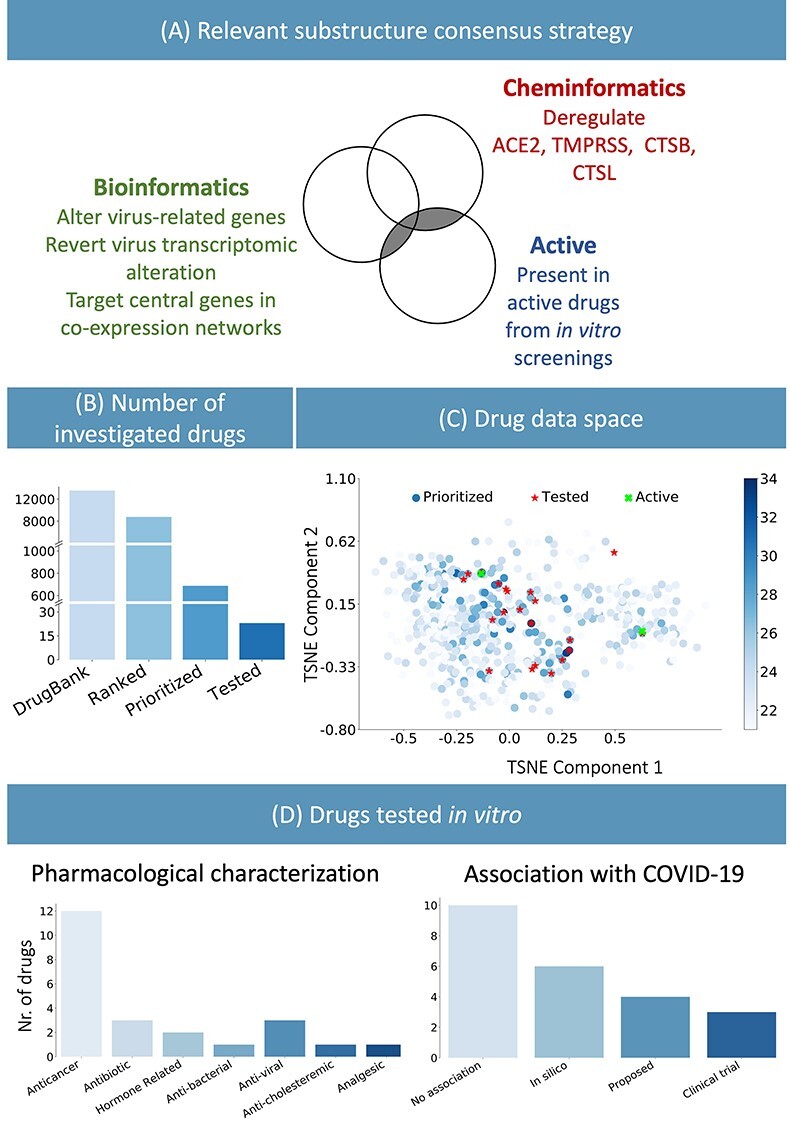
(**A**) Consensus strategy to identify relevant chemical substructure, using bioinformatics and cheminformatics methods as well as experimental results from published literature. (**B**) The suggested approach allows reducing the number of experimental tests: the whole DrugBank database was filtered to less than 800 relevant drugs and *in vitro* testing was performed on 23 candidates. (**C**) Graphical representation of the prioritized drugs. The shade blue represents the number of chemical substructures identified in (**A**), present in the drugs. The 23 selected compounds are shown in red. They were selected among the drugs sharing the most relevant substructure as well as satisfying practical logistic criteria. Of the 23 drugs, the two highlighted in green have been experimentally identified as active. (**D**) Pharmacological characterization and description of known association with COVID-19 of the 23 tested drugs. *In silico* refers to drugs derived from *in silico* studies, whereas proposed refers to drugs suggested for their potential therapeutic role in literature.

Moreover, by applying QSAR-based cheminformatics methods, we identified chemical substructures of drugs predictive of the deregulation level of the ACE2 receptor, the transmembrane protease TMPRSS2 and the cell surface proteolytic enzymes CTSB and procathepsin L (CTSL) (Figure 1**E** and **H**_2_). Finally, we retrieved chemical substructures of the drugs that were identified as active in previous screenings for COVID-19 (Figure 1**F** and **H**_3_) (the list of assays and thresholds used to define drugs as active is available in Supplementary Table S1).

We merged the three sets of chemical substructures with the aim of increasing the robustness of the predictions as well as to generate knowledge readily usable in the context of *de novo* drug development (Figure 2**A**, Supplementary Table S2). Based on the presence of these chemical substructures, we identified candidate drugs effective against COVID-19, by computationally prioritized drugs from the DrugBank database [37, 38] with PubChem-available fingerprints (Figure 2**B**) (Supplementary Table S3) expected to interfere with disease-associated biological processes. Furthermore, we evaluated how different the drug prioritization would be without the effect of the substructures derived from active drugs against SARS-CoV-2. The overall ranking of DrugBank in the two scenarios achieves a Kendall Tau rank correlation of 0.77. Thus, our integrative strategy would still result in a useful prioritization even if prior knowledge on effective drugs was not available (e.g. at the beginning of the pandemic), helping to avoid the expensive screening on large drug libraries.

### Computationally aided drug prioritization identifies a subset of candidate drugs for COVID-19

From the 8000 drugs examined from DrugBank, we focused on the top 700 to select a set of relevant candidates for further experimental validation. Our inclusion criteria was a trade-off between the selection of a set of drugs that best represents the top of the prioritized list, based on their chemical substructures (Figure 2**C**), and a number of practical considerations such as price, availability, shipping time and ease of storage. Taking into account the aforementioned criteria, we validated our method by performing an *in vitro* biological evaluation of 23 selected drugs (the drug list is available in Supplementary Table S3).

The majority of drugs currently in clinical trials for COVID-19 treatment present either antiviral or immunomodulating properties, with the aim of targeting the viral life cycle and alleviating the lung-damaging symptoms [18, 39]. Kinases play an important role in many of these biological processes, and therefore, different kinase inhibitors have been proposed for COVID-19 treatment [40, 41]. These compounds show pharmacodynamic properties allowing the dual goal of mitigating both host immunological response and antiviral activity [40]. Twelve out of the 23 identified drugs relate to oncological treatments, and eight of them act as kinase inhibitors (Figure 2**D**). Similarly to antiviral drugs, anticancer drugs may, indeed, target biological processes, which have a crucial role in modulating the organism immune response, cell division and death, cell signaling and microenvironment generation [42]. Several studies on the repurposing of anticancer drugs to treat COVID-19 already exist [42–44]. For instance, Roshewski *et al*. showed that acalabrutinib, a selective Bruton tyrosine kinase inhibitor, can mitigate the hyperinflammatory immune response characterizing the most severe cases of COVID-19 [45]. The histone methyltransferase inhibitor pinometostat, instead, seems to decrease the level of NF-kB, one of the main players of the immunological response [46], and to alleviate the host-response against infections [47]. Inhibitors of intracellular calcium homeostasis seem to block virus-induced cell–cell fusion (known as syncytia formation), one of the hallmarks of severe SARS-CoV-2 infection [44, 48].

Our analysis successfully highlighted several drugs that are either under investigation or acted effectively against SARS-CoV-2 infection. The selected 23 candidates for further investigation comprise anticancer, antimicrobial and antiviral drugs (Figure 2**D**). Drugs from all three classes showed to lower the virus titer and to tune down the cytokine storm syndrome in the most severe cases of the disease [49]. As expected, our approach identified antiviral drugs (against hepatitis C), which were already predicted as a COVID-19 treatment, or are currently in clinical trials against SARS-CoV-2 infection (https://clinicaltrials.gov/ct2/show/NCT04498936) [50, 51]. Among the three identified antibiotics, delafloxacin, a fluoroquinolone antimicrobial agent, was also studied for its antiviral activity at the early stages of the COVID-19 pandemic [52].

Altogether, approximately half of the tested candidate drugs were proposed as a potential COVID-19 treatment (Figure 2**D**). This demonstrates the capability of our methodology to identify potential drug candidates and to highlight a new set of existing compounds without previous association to COVID-19 or SARS-CoV-2.

### Validation in human cells confirmed 7-hydroxystaurosporine and bafetinib as potential COVID-19 treatment

**Figure 3:**
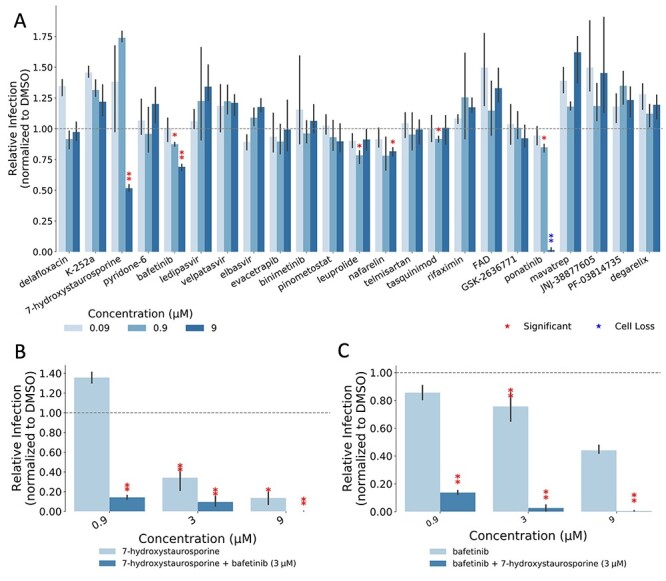
(**A**) Percentage of infected cells after drug treatments normalized by the median of the DMSO control. Each drug was added 45 min before infection and infected cells fixed 16 h later; red asterisks show significant *P-*values (<0.05) for the one-tailed *t*-test between each treatment and the DMSO. (**B**) Combined effect of 7-hydroxystaurosporine and bafetinib added 2 hbi at indicated concentrations. Cells fixed 16 hpi. (**C**) Combined effect of bafetinib and 7-hydroxystaurosporine added 2 hbi at indicated concentrations. Cells fixed 16 hpi.

To experimentally validate our computational predictions, we tested the set of candidate drugs at different concentrations (0.09, 0.9 and 9 μM) on HEK-293 T cells stably expressing human *ACE2* and *TMPRSS2* (HEK-293 T-AT) [53]. Cells were infected with the SARS-CoV-2 strain initially isolated from Wuhan (here referred as wild type, WT), at a multiplicity of infection (MOI) of 0.5 infectious units (i.u.) per cell for 16–18 h. The percentage of infected cells was determined by immunofluorescence detection of viral proteins followed by automated imaging and image analysis (Supplementary Figure S1**A**). Out of the 23 drugs, 7-hydroxystaurosporine and bafetinib showed a statistically significant inhibition of the number of virus-infected cells when tested at 9 μM, showing relative infection values of 0.51 and 0.69, respectively (Figure 3**A**, red asterisks). Ponatinib, instead, significantly inhibited the infection but also displayed a strong cytotoxic effect (Figure 3**A**, blue asterisk, Supplementary Figure S2) and was therefore excluded from further analysis.

7-Hydroxystaurosporine is an antineoplastic agent with potent *in vitro* and *in vivo* activities, and its capability of sensitizing a variety of cell lines *in vitro* was previously described [54, 55]. 7-Hydroxystaurosporine is often used in combination with other drugs for its synergistic effect of enhancing cytotoxic effect in human cancer cells, e.g. in treating leukemia (https://www.clinicaltrials.gov/ct2/results?cond=&term=UCN-01&cntry=&state=&city=&dist=) [54, 56, 57]. To our knowledge, no connection between 7-hydroxystaurosporine and SARS-CoV-2 emerged in terms of possible COVID-19 treatment. Bafetinib is a second generation tyrosine kinase inhibitor prescribed against Philadelphia chromosome-positive chronic myelogenous leukemia [58]. In addition, bafetinib was recently identified as a SARS-CoV-2 inhibitor in other drug repurposing studies [59, 60]. Bouhadduo et al. proposed and tested bafetinib as a possible COVID-19 drug, modifying the phosphoproteome of SARS-CoV-2 infected cells *in vitro* [60].

### Combination of 7-hydroxystaurosporine and bafetinib inhibits SARS-CoV-2 infection

Next, we tested whether a combination of the two identified compounds would result in stronger antiviral activity without compromising cell viability. For this, we performed two additional infection assays in which we exposed the HEK-293 T-AT cells to 7-hydroxystaurosporine and bafetinib alone, or in combination. In the first assay, the concentration of bafetinib was fixed at 3 μM, whereas the concentration of 7-hydroxystaurosporine varied as 0.9 μM, 3 μM and 9 μM (Figure 3B, Supplementary Figure S1B). In the second assay, the concentration of 7-hydroxystaurosporine was fixed at 3 μM, whereas the concentration of bafetinib varied as in the first assay (Figure 3C). The cells were exposed to the drugs 2 h before infection (hbi).

These results indicate that 7-hydroxystaurosporine had a significant inhibitory effect on viral infection already at 3 μM (Figure 3B). The combination with bafetinib resulted in an increased antiviral activity, reducing the number of infected cells by >80% (Figure 3**B**). The same applies for bafetinib, which showed a significant but moderate antiviral activity at 3 μM. The inhibitory effect of bafetinib activity was even stronger when combined with 7-hydroxystaurosporine (Figure 3**C**, Supplementary Figure S3**B**).

To test if the drugs blocked SARS-CoV-2 cell entry, which occurs during the first hour of infection [61], or a post entry step of the virus life cycle, we added the drugs 2 h postinfection (hpi) and quantified the fraction of infected cells 16 hpi (Supplementary Figure S4). We observed a similar antiviral effect in cells treated after infection, indicating that the drugs mainly inhibit a post-entry step of infection (Supplementary Figure S4).

**Figure 4:**
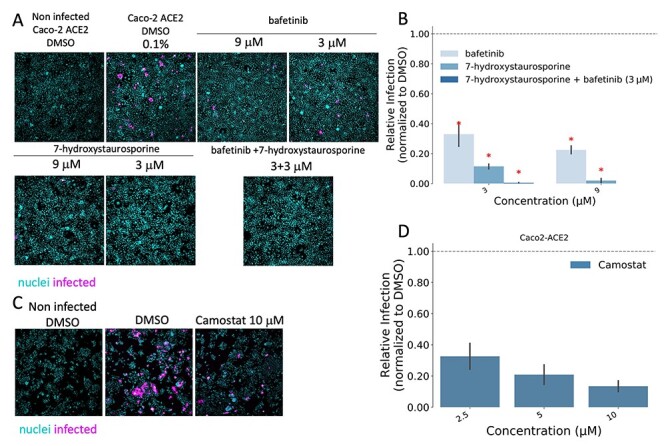
(**A**) Representative fluorescence images of Caco2-ACE2 cells treated with bafetinib, 7-hydroxystaurosporine and their combinations 2 hbi. Cells fixed 16 hpi; cyan = nuclei, magenta = infected cells. (**B**) Relative infection quantification of experiment in (**A**); values normalized to the median of DMSO controls. All values represent the averages of three experiments. Error bars indicate the SD. (**C**) Representative fluorescence images of Caco2-ACE2 cells treated with camostat. (**D**) Relative infection quantification of experiment in (**C**).

The effect of the two drugs was tested in a different cell line, Caco-2 cells stably overexpressing *ACE2* (Caco2-ACE2) and endogenously expressing *TMPRSS2* (Figure 4**A**). The antiviral effect of 7-hydroxystaurosporine, bafetinib, and of the two drugs combined, was confirmed. At a concentration of 3 μM, the infection inhibition was >60%, 80% and 90%, respectively (Figure 4**A** and **B**). As a comparison, we show that camostat, a well-known inhibitor of TMPRSS2 [62], reduces viral infection at a similar range of concentrations in Caco2-ACE2 cells (Figure 4**C** and **D**).

**Figure 5:**
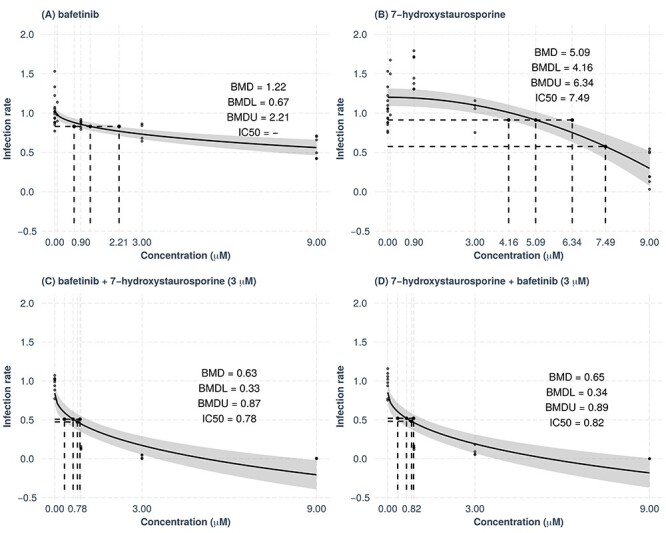
Concentration–response curve analysis. The BMD values, their lower (BMDL) and upper (BMDU) bounds and the IC_50_ values were computed for bafetinib (**A**), 7-hydroxystaurosporine (**B**) and their combinations (**C**–**D**). The *y*-axes show the infection rate normalized by the one measured in DMSO. Bafetinib and 7-hydroxystaurosporine were tested at 0.09, 0.9, 3 and 9 μM, whereas in combination they were tested at 0.9, 3 and 9 μM. The BMD, BMDL, BMDU and IC_50_ in (**C**) refer to the experiments performed where 7-hydroxystaurosporine was combined with a fixed concentration (3 μM), whereas bafetinib concentration varied.

Both bafetinib and 7-hydroxystaurosporine exhibited a concentration-dependent inhibition of viral infection (Figure 5) and predicted benchmark dose (BMD) values of 1.22 and 5.09 μM, respectively (Figure 5**A** and **B**), whereas the predicted BMD values for the two combination were 0.63 and 0.65 μM (Figure 5**C** and **D**).

HEK-293 T-AT cells, used here to validate the drug identification strategy, do not adhere strongly to imaging plates (Supplementary Figure S3**A**). Thus, cell numbers varied considerably making the estimation of drug toxicity difficult. For this reason, we next performed two separate toxicity tests, using both cell lines HEK-293 T-AT and Caco2-ACE2.

### Cytotoxicity of 7-hydroxystaurosporine and its combination with bafetinib

**Figure 6:**
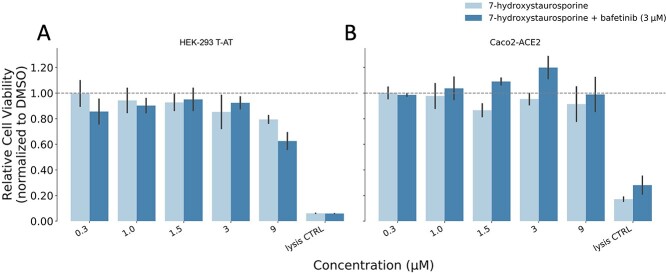
Relative cell viability in HEK-293 T-AT (**A**) and Caco2-ACE2 (**B**) cells normalized by the median of the DMSO. Cells were treated with different concentrations of 7-hydroxystaurosporine and its combination with bafetinib. Lysis buffer was also included as positive controls. All values represent the averages of four replicates. Error bars indicate the SD. Lower values of relative cell viability indicate cytotoxicity.

We determined the cytotoxicity of 7-hydroxystaurosporine alone and in combination with bafetinib using the Cello Green assay (Promega). HEK-293 T-AT and Caco2-ACE2 cells were exposed to 7-hydroxystaurosporine alone in 0.3, 1, 1.5, 3 and 9 μM, as well as in combination with bafetinib fixed to 3 μM for 20 h. No toxicity was observed in HEK-293 T-AT cells up to 3 μM and up to 9 μM in Caco2-ACE2 cells as determined by relative cell viability to dimethyl sulfoxide (DMSO) control (Figure 6).

### 7-Hydroxystaurosporine and bafetinib synergistically block SARS-CoV-2-induced cell–cell fusion

**Figure 7:**
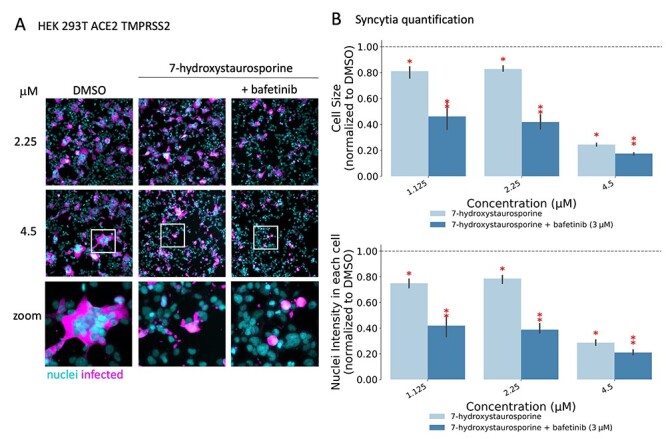
7-Hydroxystaurosporine and bafetinib inhibit virus-induced syncytia. (**A**) Representative fluorescence images of HEK-293 T-AT cells treated with indicated drugs 1 h before infection. Cells fixed 16 hpi; cyan = nuclei, magenta = infected cells. Zoomed areas from each image are indicated by white boxes. (**B**) Quantification of cell size and nuclear content from the experiment in (**A**); values normalized to the median of DMSO controls. All values represent the averages of three experiments. Error bars indicate the SD. Red asterisks show significant *P*-values (<0.05) for the one-tailed *t*-test between each treatment and the DMSO.

The ability of some pathogenic human viruses, including SARS-CoV-2, to induce cell–cell fusion, a phenomenon known as syncytia formation [63], was linked to viral spreading, pathogenicity and tissue damage *in vivo* [48]. When infected by SARS-CoV-2, HEK-293 T-AT cells efficiently fuse, forming large multinucleated cells (Figure 7**A**). In addition to a reduction in the number of infected cells, a machine learning-assisted image analysis revealed that 7-hydroxystaurosporine, at 4.5 μM, strongly inhibited virus-induced cell–cell fusion, reducing the average nuclear content per cell by >80% (Figure 7**A** and **B**, Supplementary Figure S1**C** and **D**). The combination with bafetinib increased this inhibitory effect, reducing the formation of large multinucleated cells by more than 90% and 60%, at 4.5 and 2.25 μM, respectively (Figure 7**B**). At the lowest concentration, 7-hydroxystaurosporine had a moderate inhibitory effect on syncytia formation, whereas the combination with bafetinib significantly blocked cell–cell fusion even at 1.125 μM (Figure 7**B**).

However, by targeting ABCB1 and ABCG2 transporters, bafetinib is known to increase the intracellular accumulation of anticancer drugs by blocking the drug efflux [64]. This could be a plausible mechanism for how this inhibitor enhances the effect of 7-hydroxystaurosporine. Interestingly, in addition to allowing drug efflux, this group of ABC transporters was recognized for their role in syncytialization [65]. Buchrieser *et al*. and Ou *et al*. highlighted connections between multinucleated syncytial cells and, similarly to other viruses such as measles, respiratory syncytial virus (RSV) and MERS, SARS-CoV-2 induces cell–cell fusion, a phenomenon particularly evident in severe COVID-19 [66, 67]. Sisk *et al.* demonstrated that the membrane fusion is blocked in the presence of Abl kinase inhibitors (imatinib, GNF2 and GNF5) and thus prevented syncytia formation in coronavirus (infectious bronchitis virus) spike protein-induced Vero cells [68]. More recently, another study demonstrated that the fusogenic activity of the viral spike can be inhibited by targeting cellular factors [44].

### 7-Hydroxystaurosporine and bafetinib inhibit infection of SARS-CoV-2 delta variant

**Figure 8:**
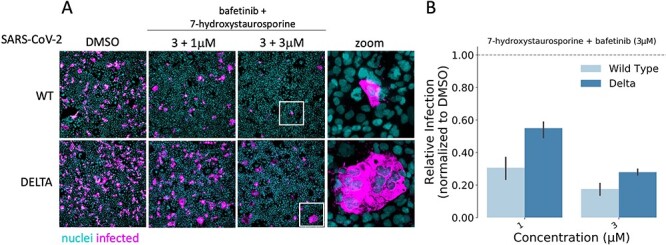
7-Hydroxystaurosporine and bafetinib inhibit delta variant infection. (**A**) Representative fluorescence images of Caco2-ACE2 cells treated with the combination of bafetinib and 7-hydroxystaurosporine 30 min before infection with WT and the delta variant. Bafetinib concentration was fixed to 3 μM, whereas 7-hydroxystaurosporine varied as 1 μM and 3 μM; cyan = nuclei, magenta = infected cells. Zoomed areas from each image are indicated by white boxes. (**B**) Quantification of relative infection from the experiment in (**A**); values normalized to the median of DMSO controls. All values represent the averages of three replicates. Error bars indicate the SD.

We tested whether the combination of 7-hydroxystaurosporine and bafetinib would also inhibit the recently emerged delta variant of SARS-CoV-2, which is known to be more infectious and more fusogenic (i.e. higher capacity to induce syncytia) than the WT strain [69]. Caco2-ACE2 cells were treated with 1 or 3 μM concentrations of 7-hydroxystaurosporine in combination with 3 μM bafetinib for 30 min before infection. The treatment also inhibited the delta variant in a concentration-dependent manner. Interestingly, and consistent with the higher infectivity and fusogenic capacity attributed to this variant, higher concentrations of drugs were required to achieve the same inhibition efficiency obtained for the WT. Even if at 3 μM the combination of the two drugs significantly decreased infection by >70%, the remaining infected cells appeared fused together (i.e. syncytia, Figure 8, zoomed images). Cell–cell fusion induced by the WT virus was almost completely inhibited at this drug concentration. These results indicate that although the combination of drugs identified in this study are also effective against SARS-CoV-2 delta variant, the dose of the drugs must be increased to achieve sufficient inhibition of infection.

**Figure 9:**
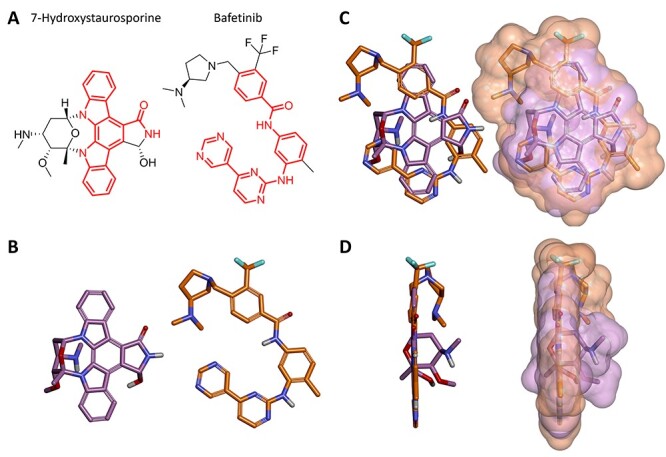
Structural comparison between 7-hydroxystaurosporine and bafetinib. (**A**) 2D structures of 7-hydroxystaurosporine (left) and bafetinib (right); the conjugated π-bond system is highlighted in red. (**B**) 3D structures of 7-hydroxystaurosporine (left) and bafetinib (right). (**C**) Front view and (**D**) side view of 7-hydroxystaurosporine and bafetinib 3D structural overlay with (right) and without (left) solvent accessible surface. Color code for (**B**), (**C**) and (**D**): carbon atoms and solvent-accessible surfaces are shown in lilac and orange for 7-hydroxystaurosporine and bafetinib, respectively; oxygen atoms in red; nitrogen atoms in blue; fluorine atoms in cyan and hydrogen atoms in white.

### Structural comparison between 7-hydroxystaurosporine and bafetinib

As 7-hydroxystaurosporine and bafetinib exhibited the highest inhibitory activity against SARS-CoV-2 infection at low micromolar concentrations, we investigated further their 2D structures and 3D geometries. In terms of molecular fingerprints, most of the common substructures highlighted aromaticity-related similarities. In fact, based on their 2D structures (Figure 9**A**), both candidates possess a highly conjugated π-bond system with 22 and 27 sp^2^-hybridized atoms for 7-hydroxystaurosporine and bafetinib, respectively. Fused aromatic rings and a high degree of conjugation suggest a planar geometry, which was confirmed when we generated and compared their 3D models (Figure 9**B**–**D**). Despite the different nature of their 2D structural scaffolds, the generated 3D geometries overlayed and displayed a 67% shape-similarity (Supplementary Table S4), which may partially explain their antiviral activity in the biological assays. Although we cannot suggest a common target, both compounds are known to bind several proteins and induce multiple downstream effects (Supplementary Table S5). The 3D conformation generated for bafetinib is in line with the model presented by Zhang *et al*. [64], with the ligand docked into the binding pocket of the ABCG2 transporter efflux. Inhibition of ABCG2 also supports the possible explanation for the synergistic effect when the compounds were used in combination. However, as 7-hydroxystaurosporine and bafetinib lowered the infection rate also when tested alone, a clear explanation for their intrinsic antiviral activity remains unknown. Of note, the drug candidate K-252a shares the same aromatic and planar core of 7-hydroxystaurosporine (Supplementary Figure S5); however, it did not show inhibitory activity in the biological assays. The lack of its intrinsic activity may be due to the missing hydroxy group in the isoindolinone moiety and/or to the different substituents (e.g. the lack of a basic functional group) and the number of the sp^3^-hybridized carbon atoms in the conformationally distinct aliphatic heterocyclic moieties i.e. tetrahydro-2*H*-pyran and tetrahydrofuran in 7-hydroxystaurosporine and K-252a, respectively. In addition, we used the webtool (https://bqflab.github.io) WADDAICA [70] to assess whether bafetinib and 7-hydroxystaurosporine would contain any structures associated with pan-assay interference compounds (PAINS) [71], thus reducing the impact of our results. No associations to PAINS were detected for either bafetinib or 7-hydroxystaurosporine.

## Conclusive remarks

One of the lessons learned during the current pandemic is that innovative approaches are required to speed up drug development while increasing its success rate. Large experimental screenings are currently used to identify potential leading compounds, but they require significant time and resources.

We computationally prioritized the DrugBank database for potential SARS-CoV-2 inhibitors by using an integrated procedure comprising multiple bioinformatics and cheminformatics methods. Our strategy allowed us to make an informed decision about which drugs to test, out of almost 8000 drugs from DrugBank, thus significantly reducing the potential costs of drug repositioning, while simultaneously increasing the success rate. We experimentally assayed 23 representatives of the prioritized library and found that two drugs, 7-hydroxystaurosporine and bafetinib, showed significant inhibition of viral infection. In addition, an image analysis of the infected versus treated cells showed that the formation of multinucleated syncytial cells was also significantly reduced. Unexpectedly, when combined, the two drugs exerted an even stronger, synergistic inhibition of viral infection as well as cell–cell fusion inhibition at lower concentrations. Further *in vitro* experimentation showed that the drugs in combination were still effective 1 h after the infection of the cells, suggesting that they may hinder a post entry mechanism of the virus. Moreover, our results also confirmed the effectiveness of the combination of the drugs against the more infective SARS-CoV-2 delta variant.

Even though the focus of this work is on the integrated computational methodology, we speculated that a possible synergistic mechanism is based on the inhibitory effect of the ABCG2 transporter efflux of bafetinib, which could potentially increase the intracellular concentration of 7-hydroxystaurosporine and therefore potentiate its effect. However, further experimental work for characterizing the MOA of the drugs could be performed in follow-up studies.

Our integrated approach can be generalized by including other types of prior knowledge to guide drug prioritization and it could significantly help the repurposing of drugs for other diseases. In addition, our results are not limited to candidate repositionable drugs but include a characterization in terms of chemical substructures relevant to the viral system considered. Indeed, we conjecture that this set of chemical substructures can be further exploited in the context of scaffold-based *de novo* drug design.

## Methods

### RNA-Seq data preprocessing

Human transcriptomics datasets analyzed in this study were retrieved from the Gene Expression Omnibus (GEO) repository, annotated with the GEO ID GSE147507 [72, 73]. The datasets are composed as following: human lung biopsies of SARS-CoV-2-infected patients and uninfected control; A549 cell line infected with SARS-CoV-2, A549 cell line infected with SARS-CoV-2 overexpressing ACE2, Calu-3 cells infected with SARS-CoV-2; NHBE cell line infected with SARS-CoV-2. For each of the cell lines, the mock treated lines were collected to be used as controls for the expression analysis. The preprocessing of human transcriptomics datasets was carried out starting from the raw counts provided within the GEO record. To remove the less biologically relevant features, low read counts were filtered by applying the proportion test method implemented within the NOISeq Bioconductor package [74]. The proportion test is a procedure that allows to identify genes expressed at levels higher than a given cut-off (see NOISeq documentation for more details), by assigning a P-value to each feature. Features with *P*-value > 0.05 in all the biological conditions are discarded. Filtered counts were then normalized through the median of ratios method implemented in the DESeq2 package [75] to make the samples comparable for differential analysis [76]. Median of ratios normalization takes into account the sequencing depth and the RNA composition of the samples to be compared, so it is considered a standard method for between-samples comparisons. Differential expression analysis was carried out by using the DESeq2 Bioconductor package and the *P*-values were adjusted using the Benjamini–Hochberg method [77].

### Co-expression network inference and analysis

Five co-expression networks were inferred for both the human biopsies and all the infected cell lines by using the clr algorithm [78] implemented in the minet package [79] with Pearson correlation as the estimator. The expression values of the DEGs were used to infer the networks. The subsequent operations on the inferred networks were carried out through the use of the INfORM tool [80]. INfORM is a software designed to carry out the most common operations on (biological) networks, such as network inference, network-based gene prioritization, module detection, functional annotation. In this work, we exploited INfORM functionalities to get a robust network-based gene rank for each of the networks. The robustness of the gene ranks is ensured by a multi-rank aggregation function, implemented in INfORM, that is based on several centrality measures, including degree, closeness, clustering coefficient, betweenness and eigenvector and a biological significance score calculated as abs(log_2_FC × −log_2_(*P*-value)), where FC is the fold change of the genes and *P-*value is the significance of the differential expression analysis. In detail, INfORM builds gene ranks for each of the aforementioned metrics and then it aggregates them through the use of the Borda function, implemented in the TopKLists package [81].

### Open TG-GATEs data preprocessing

Raw microarray data for 129 drugs were downloaded from the Open Toxicogenomics Project-Genomics Assisted Toxicity Evaluation System (TG-GATEs) repository (https://dbarchive.biosciencedbc.jp/en/open-tggates/download.html) [82]. The dataset comprises *in vivo* samples from the liver and kidney of rats exposed to three dose levels of the drugs at four time points as well as a respective set of *in vitro* samples from rat primary hepatocytes. The samples were imported in R by using the justRMA function [83] from the R library Affy [84]. Outliers were identified with the RLE and NUSE functions from the affyPLM package [85] and the slope of the RNA degradation curve implemented in the affy package [84]. A sample was removed from the analysis when marked as outlier by at least two out of the three methods. The probes were annotated to Ensembl genes [by using the rat2302rnensgcdf (v. 22.0.0) annotation file] from the brainarray website (http://brainarray.mbni.med.umich.edu/), and the resulting expression matrix was quantile normalized by using the normalizeQuantile function from the limma package. In the end, only the subset of *in vivo* samples from rat liver with a single exposure experimental setup were considered for further analysis, and the ensembl genes were mapped to gene symbols by using the AnnotationDBi package [86].

### Dynamic dose-responsive PODs

For each drug of the Open TG-GATEs, the dynamic dose-dependent MOA and the corresponding POD were identified through the use of the TinderMIX software [87]. Starting from the pairwise log_2_ fold change (log_2_FC) of each gene (computed as the difference between the log_2_ expression values of each pair of treated and control samples), 1st-, 2nd- and 3rd-order polynomial models were fitted. For the best-fitting model, its contour plot was computed as an effect map. Next, the contour plot of each gene was evaluated to identify an area showing a dynamic dose–response i.e. an area where the expression changes monotonically in respect to the dose once an activity threshold has been reached. As for the classical BMD analysis, an activity threshold of 10% was selected [88–90]. If such an area was identified, the gene was considered to be altered in a dynamic dose-dependent manner.

The genes were then labeled with an activation label that specifies its POD based on the lowest dose and the earliest time of activation. The time-dose effect map was divided into a 3 by 3 grid and the sections of the dose axis were named ‘S’ (sensitive), ‘I’ (intermediate) and ‘R’ (resilient), whereas for the time axis, the labels ‘E’ (early), ‘M’ (middle) and ‘L’ (late) were assigned. The final label was then obtained by identifying the earliest and most sensitive point of activation and concatenating the dose and time of the single labels.

### Drug prioritization strategies

#### Dose-dependent SARS-CoV-2 physical interactors

The list of physical interactors with the SARS-CoV-2 was retrieved from Gordon *et al*. [26]. This list of proteins was translated into human gene symbols using the R biomaRt package [91]. In this article, they are further referred to as the Gordon’s genes. The Gordon’s genes were mapped to the *Rattus norvegicus* ortholog genes using the R biomaRt package [91, 92]. Then, for each drug in the Open TG-GATEs, a score was computed by summing the number of Gordon’s genes that were considered dynamic dose responsive by the TinderMIX analysis and the strength of deregulation as the sum of their log_2_FC. The log_2_FC of a dynamic dose-responsive gene was computed as the mean log_2_FC of its dynamic dose-responsive area [87]. The drugs were ranked according to this score from the highest to the lowest.

#### DEGs in SARS-CoV-2 samples

Similarly, the DEGs identified for the human lung biopsy of SARS-CoV-2 patient, the A549, Calu-3 and NHBE cell lines infected with (SARS-CoV-2) and the A549 cell line infected with SARS-CoV-2 overexpressing *ACE2* were mapped to their corresponding rat orthologs. Then, for each drug in the Open TG-GATEs, a score was computed by summing the number of DEG identified as dynamic dose responsive by the TinderMIX analysis in each SARS-CoV-2 condition and their strength of deregulation. This resulted in five different ranks of the Open TG-GATEs drugs, where the drugs that strongly deregulate the same DEG of each SARS-CoV-2 condition are at the top of the list.

#### Connectivity mapping

For each one of the SARS-CoV-2 conditions, the genes were ranked from the most upregulated to the most downregulated. For each drug of the Open TG-GATEs, the dynamic dose-responsive genes identified by the TinderMIX analysis were divided into two groups depending on whether their log FCs were monotonically increasing or decreasing in respect to the dose.

The gene set enrichment analysis (GSEA), based on the Kolmogorov–Smirnov test [93], was used to compute the pairwise similarity between the Open TG-GATEs drugs and the SARS-CoV-2 conditions. The Kolmogorov–Smirnov test can be used to compare a sample with a reference probability distribution. The Kolmogorov–Smirnov statistic was used without the absolute value to preserve the sign [94]. This helps to understand if the increasing and decreasing dynamic dose-responsive genes derived from the Open TG-GATEs drugs are up- or downregulated in the SARS-CoV-2 conditions. Thus, for each of the five SARS-CoV-2 conditions, the Open TG-GATEs drugs were ranked based on their capability to reverse the transcriptomic alterations due to the SARS-CoV-2 infection weighted by the GSEA statistics.

#### Drug targets and co-expression analysis

All data annotated in the OpenTargets database [95] were retrieved as a compressed JSON file. These data contain the drug–targets associations used in this study. The targets were mapped on the five co-expression networks, and their aggregated ranks from the INfORM [80] strategy were retrieved. The drugs were ranked according to the median rank of their corresponding targets. In this way, drugs whose targets are more central to the network are ranked at the top of the list.

#### Drug ranking with Borda

To summarize, one ranking was produced by the dose-dependent analysis of the physical interactor alterations, whereas the analyses of the DEGs, the connectivity mapping and the drug targets produced five rankings each, one for each SARS-CoV-2 condition. The 16 lists of ranked drugs were merged together by using the Borda method [81] implemented in the R package TopKLists. In this way, a final consensus on the drug ranks was identified.

### Relevant chemical substructures

A GSEA analysis was performed to identify the presence of statistically enriched chemical substructures in the drugs ranked at the top of the list. A binary matrix with 881 chemical substructures for the drugs was created. A drug was assigned to the list of a specific substructure if that drug contains the substructure. A substructure was considered statistically enriched if the *P*-value of the GSEA was lower than 0.05.

### QSAR

#### Data preprocessing

A QSAR analysis [96] was performed on the Open TG-GATEs drugs using the pairwise levels of differential expression of a chosen set of genes as the response variable. Namely, *ACE2*, *TMPRSS2*, *CTSB* and *CTSL* were used. For each gene, the expression levels at all dose levels and time points were considered simultaneously. The initial dataset for each gene comprised the pairwise differential expression levels at all the doses and all the time points together with a set of dummy variables to represent the dose levels and the time points. Substructure fingerprints for the Open TG-GATEs drugs were retrieved from PubChem [97] by querying it by Compound ID (CID) identifiers. Each differential expression level was represented by the set of binary fingerprints retrieved by PubChem plus seven indicator variables: three to represent the dose levels and four to represent the sacrifice periods. Each of the 881 bits forming the fingerprint indicate the presence or absence of particular chemical substructures, ranging from counts of single atoms, to the presence and the type of bonds between atoms, to more complex substructures like aromatic rings [98].

Since the fingerprint data matrix was sparse, it was preprocessed by removing all the fingerprints absent in any drug of the dataset manipulating the data matrix with the python module pandas [99]. After this step, the number of variables to use in the modeling phase was further reduced by evaluating the correlation coefficient between each pair, and, to obtain a valid distance metric among the features, the correlation matrix C was transformed according to $\sqrt{1-C^{2}}$ [100]. DBSCAN [101] clustering implemented in the python module scikit-learn [102] was then applied with parameters epsilon = 0.1 and the minimum number of samples as 2. In this way, an automatic grouping of the most correlated variables was obtained. Finally, each cluster of correlated variables is compressed into one. The resulting data matrix was reduced to about 400 variables.

#### Modeling

After preprocessing, the data were modeled using a gradient boosting machine [103] from the scikit-learn module [102].

Gradient boosting fits a model F(x) built as the additive combination of an ensemble of functions ${f}_i(x)$ for $i=1,\dots, m$ belonging to a chosen functional class, known as base or weak learners. Commonly used weak learners are the classification and regression trees [103]. Weak learners are added sequentially in a stagewise manner:

$$ {F}_0(x)={w}_0\cdotp{f}_0(x) $$

$$ {F}_1(x)={F}_0(x)+{w}_1\cdotp{f}_1(x) $$

$$ {F}_2(x)={F}_1(x)+{w}_2\cdotp{f}_2(x) $$

$$ \cdots $$

$$ {F}_m(x)={F}_{m-1}(x)+{w}_m\cdotp{f}_m(x) $$

Each stage improves or ‘boosts’ the loss $L(y,{F}_m(x))$by adding a weak learner ${f}_{m+1}(x)$ fitted to the functional gradient of the loss function of the model up to the *m*-th step, whereas each weight ${w}_i$ is fitted by a line search algorithm [103]. Common loss functions used for regression are the squared loss

$$ {L}_S\left(y,{F}_m(x)\right)=\sum \limits_i{\left({y}_i-{F}_m\left({x}_i\right)\right)}^2 $$

and the absolute loss

$$ {L}_A\left(y,{F}_m(x)\right)=\sum \limits_i\mid{y}_i-{F}_m\left({x}_i\right)\mid $$

where ${({x}_i,{y}_i)}_{i=1,\dots, n}$ is the training set.

Both losses present advantages and disadvantages that make the choice of the loss dependent on the dataset at hand. For example, the squared loss ${L}_S$ has a well-defined gradient function (namely the residual errors of predictions) that makes the fitted models stable across repetitions; however, squaring the errors puts a lot of emphasis on large errors. This implies that models fitted using ${L}_S$ may be more susceptible to noise. On the other hand, the absolute loss ${L}_A$ puts less emphasis on large errors, thus making models more robust to noise; however, the discontinuity of the loss is detrimental to the stability of the models.

Indeed, in preliminary experiments (data not shown) we observed that when fitting models with ${L}_A$ led to more variance in the estimation of the variable importance, also, the predictions of extreme deregulation values were less precise, due to the lower emphasis on large errors. Thus, we decided to keep the ${L}_S$ loss and only optimize the rest of the hyperparameters with a grid search.

The number of estimators was fixed to 500 for computational constraints and instead we explored different regularization parameters configurations. A grid of possible parameter settings was explored using a 5-fold cross-validation repeated 25 times for each combination of parameters. The grid of parameters was defined as follows:

- subsample, the proportion of samples used to train each individual tree ranged in [0.2, 0.8],

- max_features, the number of features to evaluate at each node ranged in [1, 50],

- learning_rate, the magnitude of the learning rate varied between [0.001, 0.1],

- max_depth, the maximum height of each decision tree ranged in [1, 5],

- criterion, the function used to evaluate each split was one of {‘mse’, ‘mae’, ‘friedman_mse’}.

Performances of each fitted model were evaluated on the corresponding held-out fold. Due to the lack of an external validation dataset, only internal validation of the fitting was performed. For this reason, the parameters corresponding to the most regularized (i.e. parsimonious) models within 1 *SD* from the minimum error achieved were chosen [104].

The best model parameters were identified as subsample = 0.5, learning_rate = 0.1, max_features = 8, criterion = ‘mse’, max_depth = 3, which resulted in a trade-off between validation accuracy, regularization and computing time. The best performing models had a validation root-mean-square error loss of 1.5 ± 0.6.

When the most appropriate parameters were selected for each gene, the best models were fit again using the whole dataset to obtain the final predictors. Since each drug fingerprint representation appears more than once in the dataset due to the different sacrifice periods and dose levels, care must be taken when considering splits of the dataset into train and validation sets. To this end, every model trained in these experiments was fit and evaluated on datasets split at the drug level, meaning that all instances of a drug are in either the training or validation sets. This is to avoid any information leakage that could happen when some of the dose levels or time points of the same drug are split across the training and validation sets.

#### Chemical substructure relevance

After fitting, each model was exploited to identify the most relevant fingerprints, which, on average, are mostly associated with the over- or underexpression of the analyzed genes. To this end, the partial dependence [103] of the predicted outcome on each fingerprint was computed. Since the optimal selected models fitted decision trees with a maximum depth higher than 1, feature interactions made the ranking slightly unstable, so the fit was repeated for each model 250 times and the relevance of each molecular substructure across the runs was averaged. Finally, each feature was ranked based on its contribution to the average predicted level of differential expression. Each molecular substructure was considered as relevant for under (resp. over) expression if the 75th (resp. 25th) percentile of the partial dependence of the response variable is lower (resp. higher) than 0.

### Chemical substructures in screened drugs

A dataset of 6975 chemical compounds screened for activity against several cytotoxicity endpoints from the literature was collected [23–26]. The included articles are reported in Supplementary Table S1. For each considered pair of drug and endpoint, an activity threshold was defined in the same way as in the original articles. These activity thresholds were used to define an activity binary variable for each screened drug. The same kind of fingerprints of chemical substructures were also collected for this dataset and a χ^2^ statistical test was performed on each chemical substructure feature against the activity variable to identify the chemical substructures statistically relevant (*P*-value < 0.05) to the activity threshold.

### Drug prioritization

The DrugBank database v. 5.0 was retrieved for this study. DrugBank [37, 38] contains 13 579 drug entries, including 2635 approved small molecule drugs, and over 6375 experimental drugs. The DrugBank IDs were matched with the PubChem [97] CID using the PubChem Identifier Exchange Service (https://pubchem.ncbi.nlm.nih.gov/idexchange/idexchange.cgi). For the 8775 matched compounds, substructure fingerprints were retrieved from PubChem by querying it by CID identifiers.

To determine the overall ranking of the DrugBank dataset, we defined the final set of relevant substructures as the union of the fingerprints derived from each single bioinformatics and cheminformatics approach. We further constrained the overall set of relevant substructures by considering its intersection with the set of relevant substructures derived from the screened drugs found in the literature. This resulted in a set of 53 relevant molecular substructures. The drugs were ranked according to the Tanimoto similarity [105] with respect to the molecular substructures relevant to the models and considered as possible candidates for further investigation.

### Structural comparison of 7-hydroxystaurosporine and bafetinib

The structures of 7-hydroxystaurosporine and bafetinib (Figure 9) were generated in three dimensions and minimized by applying the MM2 energy-minimization method in ChemBio3D (ChemDraw^®^ Professional v20, PerkinElmer Informatics, Inc.). The minimized structures were overlaid by steric fields and the similarity (Supplementary Table S4) was calculated in Discovery Studio Visualizer v21.1 (Dassault Systèmes Biovia Corp).

### Image analysis for syncytia quantification

The fluorescent images (2048 × 2048 pixels) of cells stained with the nuclear dye were used to train a deep convolutional neural network to segment first the nuclei and then the entire cell using the fluorescent signal of the N immunostaining. For the training, we first used 18 images that, according to visual inspection, faithfully represented the variation (cell size, morphology, average intensity) of the whole dataset. We then randomly extracted a smaller area of 1024 × 1024 sized from each image, and normalized the intensities by dividing them with the global upper bound of the intensities found in the original dataset and converted them to 8-bit format. These selected images were used to manually segment the contour of each N-labeled cell. After this fast initial manual annotation, we utilized image augmentation techniques to prevent the model from overfitting. We created an augmentation pipeline containing seven main transformations where each transformation instance is applied with a probability of 0.5 implemented using the Numpy scientific programming library and the Pillow package (https://pillow.readthedocs.io/en/3.0.x/reference/ImageEnhance.html) for Python [106]. Some of the transformations have input parameters; in this case, the parameter is sampled from an interval divided by step size of 0.01. The transformations used in our augmentation pipeline are summarized in Supplementary Table S6. We constructed 100 augmented instances of each image, and therefore, we have (100 + 1) × 18 = 1818 images in the extended training set. We then trained a popular TensorFlow [107] implementation (https://github.com/matterport/Mask_RCNN) of the Mask Region Based Convolutional Neural Network (R-CNN) instance segmentation algorithm [108], to detect the cell instances with transfer learning by fine tuning a previous cytoplasm segmentation model to this task. We trained the model until convergence through 13 epochs (full model 10 epoch and 1 epoch for the layers 4+, 3+ and heads), where each epoch contains 2000 steps with batch size of 1. In the Mask R-CNN training we defined the non-max suppression probability to 0.55 (for the RPN training) and the detection minimum confidence threshold to 0.5, whereas the non-max suppression threshold (during the detection) was 0.35. We used a stochastic gradient descent optimizer with learning rate 0.001 and momentum 0.9.

### Cell culture

HEK-293 T stably expressing human *ACE2* and *TMPRSS2* (HEK-293 T-AT) have been previously described [53]. Caco-2 cells stably expressing human *ACE2* (Caco2-ACE2) were generated by transduction with third generation lentivirus pLenti7.3 ACE2-EGFP, where the expression of *EGFP* is guided by a separate promoter downstream of the *ACE2* coding sequence [53]. EGFP positive cells were isolated by FACS sorting. All cells were grown in Dulbecco's modified Eagle's medium (DMEM) media supplemented with 10% fetal calf serum (FCS), pen/strep, L-glutamine and passaged 1:10 (HEK-293 T-AT) or 1:6 (Caco2-ACE2), every 3 days.

### SARS-CoV-2 infection

All experiments with WT (strain B.1) and delta variant (strain B.1.617.2) SARS-CoV-2 were performed in BSL3 facilities at the University of Helsinki with appropriate institutional permits. Virus samples were obtained under the Helsinki University Hospital laboratory research permit 30 HUS/32/2018 § 16. The virus was propagated once in Calu-1 cells and once in VeroE6-TMPRSS2 cells (WT) or once in VeroE6-TMPRSS2 cells (delta) before sequencing and storage at −80°C. Virus stocks were stored in DMEM, 2% FCS, 2 mM L-glutamine, 1× pen/strep as previously described. [53] Virus titers were determined by plaque assay in VeroE6 TMPRSS2 cells. For testing small molecule inhibitors, cells in DMEM, supplemented with 10% FBS, 1× GlutaMax, 1× pen/strep20, mM HEPES pH 7.2 were seeded in 96-well imaging plates (PerkinElmer Cat. No. 6005182) 48 h before treatment at a density of 15 000 cells per well. Drugs or DMSO control, were either added 60 min before infection, or added 90 min postinfection. Cells were infected at a MOI 0.5 plaque forming units per cell (titer determined in VeroE6-TMPRSS2 cells). Infections were carried out for 20 h in a 37°C and 5% CO_2_ incubator. Cells were then fixed with 4% paraformaldehyde in phosphate-buffered saline (PBS) for 30 min at room temperature before being processed for immunodetection of viral N protein, automated fluorescence imaging and image analysis.

### Immunofluorescence, imaging and image analysis

Fixed cells were washed once with Dulbecco PBS containing 0.2% BSA (D-BSA), and permeabilized for 10 min at room temperature in the same buffer containing 0.1% Triton X-100 (W/V, Sigma) and 1 μg/ml Hoechst DNA staining (Thermo Fisher, Cat. No. H3570). After one wash in D-BSA, cells were incubated for 1 h at room temperature with a 1:2000 dilution of a polyclonal rabbit antibody raised against the viral N protein of SARS-CoV that cross-reacts with the N protein of SARS-CoV (a kind gift of Prof. Ilkka Julkunen, University of Turku, Finland. [109]). This antibody cross-reacts with SARS-CoV-2 N protein. Following two washes in D-BSA, cells were incubated with a fluorescently labeled goat anti-rabbit antibody (Molecular Probes) at a dilution of 1:1000 for 1 h at room temperature. After two washes in PBS, cells were either stored in the same buffer at 4°C or imaged directly with a Molecular Device Nano high-content microscope using a 10× objective. To determine the percentage of infected cells, the CellProfiler 3 open source software was used (www.cellprofiler.org). Nuclei stained with Hoechst were detected using the Otsu algorithm of the CellProfiler3, and infected cells identified based on fluorescence intensity of immunostained N in the perinuclear area of each cell, using a threshold of fluorescence empirically determined such that <0.01% of non-infected cells were detected as positive. The relative number of infected cells was calculated by dividing in each well the number of N-positive nuclei by the total number of Hoechst-positive nuclei.

### Quantification of inhibition

Image handling, quantification and analysis of fluorescence images were performed as aforementioned using Cell profiler 3 or a custom made machine learning algorithm. For each experiment, nine images were acquired and >2000 cells analyzed. Each experiment was repeated three to four times and values indicated in each figure represent the average and SD of all repetitions. Analysis of significance was performed using a one-tailed *t*-test to identify drugs with an infection rate lower than the DMSO. One asterisk = *P* < 0.05; two asterisks = *P* < 1e-5 after Bonferroni correction.

### Toxicity assays

HEK-293 T-AT and Caco2-ACE2 cells were seeded in 96-well imaging plates (PerkinElmer) 48 h before treatment at a density of 15 000 cells per well. Cells were treated with different concentrations of 7-hydroxystaurosporine and bafetinib for 20 h. DMSO was used as a negative and lysis buffer (CellTox Green) as a positive control. Cytotoxicity was measured using CellTox Green and CellTiterGlo2.0 assays (Promega) and Hidex Sense Microplate Reader (Hidex) according to the instructions of the manufacturer.

### Drug concentration-dependent virus inhibition analysis

A dose-dependent analysis was performed with the strategy implemented in the BMDx tool [90], to test if bafetinib, 7-hydroxystaurosporine and their combination reduce the viral infection rate in a dose-dependent manner. For the analysis, the benchmark response was set to 10% difference with respect to the controls. The linear, power, exponential and hill functions were fitted to the data. The optimal fitting model was selected as the one with the lowest Akaike information criteria and used to estimate the BMD, the corresponding lower and upper bound (BMDL and BMDU) values and the half maximal inhibitory concentration (IC_50_) value. The number of replicates used in the concentration-dependent analyses varied from 3 to 15 depending on the concentration level.

#### Supporting Information

Supporting Information is available from the Wiley Online Library or from the author.

**Key Points**

- Integrated cheminformatics and bioinformatics approaches can help to identify a subset of relevant chemical substructures for drugs active against COVID-19.
- Prioritization of the DrugBank database identifies a subset of candidate drugs for COVID-19.
- *In vitro* validation confirmed 7-hydroxystaurosporine and bafetinib as potential COVID-19 treatment.
- Combination treatment with 7-hydroxystaurosporine and bafetinib reduces SARS-CoV-2-induced syncytia.
- Combination treatment with 7-hydroxystaurosporine and bafetinib also reduces delta variant infection.

## Supplementary Material

Supplementary_materials_bbab507

Table_S3_bbab507

Table_S5_bbab507

## Notes

**Angela Serra** is a senior postdoctoral researcher at FHAIVE (Finnish Hub for Development and Validation of Integrated Approaches) at the University of Tampere. She holds a master’s degree in Computer Science (2013), and she obtained the Ph.D. in Computer Science and Information Engineering with a dissertation on multi-view learning and data integration approaches for multi-omics data (2017). Her current research focuses on data modelling, machine learning, network inference, data integration for toxicogenomics, bioinformatics and cheminformatics.

**Michele Fratello** is a postdoctoral researcher at FHAIVE (Finnish Hub for Development and Validation of Integrated Approaches) located at the University of Tampere. He has a master’s degree in Computer Science (2013), and he obtained the Ph.D. in Medical, Clinical and Experimental Sciences with a dissertation on multi-view learning in neuroimaging (2016). His current research focuses on machine learning, chemical data modelling and analysis of toxicogenomic data.

**Antonio Federico** is a Postdoctoral researcher at the Finnish Hub for Development and Validation of Integrated Approaches (FHAIVE) - Tampere University (Finland). He is specialized in computational analyses of high-throughput sequencing data and systems biology modelling in human diseases. The topic of his research is the integration of molecular networks to uncover the mechanisms of druggability in human diseases for the development of novel pharmacological therapies.

**Ravi Ojha** is a PhD student in Dr. Giuseppe Balistreri’s research group at the Faculty of Medicine of the University of Helsinki (Finland). His work focuses on understanding the mechanisms of SARS-CoV-2 and Respiratory Syncytial virus entry into human cells. He is qualified to work in BSL3 facilities, and routinely uses automated imaging techniques as well as classic cell biology and biochemical approaches.

**Riccardo Provenzani** (MSc, University of Pavia, Italy, 2012) is a medicinal chemist with over ten years of experience in international and multidisciplinary research teams. He joined the Doctoral Programme in Drug Research (University of Helsinki, 2014) working on the project “New Protein Kinase C Modulators for Cancer and Alzheimer’s Disease”. He authored 9 publications in peer-reviewed journals in the areas of medicinal chemistry, pharmaceutical technology, and microfabrication. He delivered presentations on his research at several international events including the conferences “Protein Kinase C Signalling in Health and Disease” (Dublin, June 2014) and multiple EFMCs (2015, 2017–19).

**Ervin Tasnadi** is a PhD student in computer science at the University of Szeged and works on single cell segmentation in 2D and 3D with classical and deep learning methods under the supervision of Dr. Peter Horvath at the Biological Image Analysis and Machine Learning Group, Biological Research Centre, Szeged.

**Luca Cattelani** is a computer scientist with a Bachelor Degree and a Master Degree in computer science from the University of Bologna, and a PhD from the University of Brescia. His main research focus is artificial intelligence for health, with contributions spanning Bayesian particle filters, predictive models for adverse health events, deep neural networks, bioinformatics, quantitative in vitro to in vivo extrapolation, and multi-view and multi-objective optimization with omics data. He has previously worked at the Universities of Bologna, Milano Bicocca and Tampere, and is currently working at the University of Eastern Finland.

**Giusy del Giudice** is Ph.D. student at FHAIVE (Finnish Hub for Development and Validation of Integrated Approaches), a research institute part of the Faculty of Medicine and Health Technology at the Tampere University. She holds a BSc in biotechnology for Human Health (2017) and a M.Sc. in medical biotechnology (2019). Her areas of expertise are the analysis and modelling of toxicogenomics and pharmacogenomics data, and systems immunology.

**Pia A.S. Kinaret** has degrees in biotechnology (2009), bioinformatics (2012) and genetics (2017). Her areas of expertise include laboratory and medical diagnostics, in vitro methods, toxicology, immunology and molecular biology. She receives funding from Orion Research Foundation sr.

**Laura A. Saarimäki** is a PhD student at The Finnish Hub for Development and Validation of Integrated Approaches (FHAIVE) located at the Faculty of Medicine and Health Technology, Tampere University, Finland. She combines her background in biomedical technology and molecular biology with bioinformatics for a comprehensive approach to toxicology and biomedical research.

**Alisa Pavel** is a Ph.D. student at FHAIVE (Finnish Hub for Development and Validation of Integrated Approaches) at Tampere University. She holds a M.Sc. in Computer Science (2019) from the University of Edinburgh. Her areas of expertise are biological network modelling and their analysis, as well as big data modeling for toxicogenomics and pharmacology.

**Suvi Kuivanen** is a postdoctoral researcher in Viral Zoonoses Research Unit at the University of Helsinki. She is specialized in biosafety level 3 work, and her research is focused on pathogenesis of neurotropic zoonotic viruses. Currently, the focus is on drug repurposing and inhibitor screens against SARS-CoV-2 virus infection in vitro.

**Vincenzo Cerullo, Vince** is professor of Biological Drug Development, Faculty of Pharmacy, Head of Drug Research Program, HiLIFE fellow, Translational Immunology Programme, University of Helsinki. He has been awarded the Outstading Young Investigator award in 2014 by the European Society of Gene and cell Therapy and the Excellence in Research award by the American Society of Gene and Cell Therapy in 2015; he has been recently ranked number fifth globally, and number one in Europe in his own expertise (oncolytic virotherapy) by expertscape. He has received two ERC grants and funded a successful spinoff company.

**Olli Vapalahti** is professor of Zoonotic Virology at Faculties of Medicine and Veterinary medicine of University of Helsinki (UH) and leads the UH Viral Zoonoses Research Unit. The research targets zoonotic and emerging viruses from molecular to population level and involves Biosafety level 3 work and metagenomic approaches for virus discovery. Recently the research has been largely refocused to SARS-CoV2-related projects, including molecular epidemiology, in vitro and in vivo infection models and drug repurposing screens.

**Peter Horvath** (m) (Director, Biological Research Centre, Szeged, Hungary, and Group Leader, Institute of Molecular Medicine, FIMM-EMBL, Finland) develops machine learning-driven single cell analysis techniques and combines with genomics and proteomics. His groups focus on finding computational solutions to single cell biological problems, combining wet-lab and light microscopy with image analysis and machine learning.

**Antonio Di Lieto** is MD, Ph.D. specialist in psychiatry. He obtained his Ph.D. in Behavioural science and learning processes. He has previously researched the biological correlates of psychiatric syndromes and molecular pharmacology. He is currently working as senior consultant psychiatrist at the Department of Forensic Psychiatry of the University of Aarhus, Denmark.

**Jari Yli-Kauhaluoma**, PhD is the vice-dean for research at the Faculty of Pharmacy, University of Helsinki. His research team focuses on medicinal chemistry of novel antimicrobial, especially anti-biofilm, agents as well as modulators of protein kinases, and sustainable pharmacy and drug development. He is the member of Pharmacopoeia Committee of the Finnish Medicines Agency and the Finnish Academy of Science and Letters. The current project funding is from the Academy of Finland, European Union (IMI2), Jane and Aatos Erkko Foundation, Strategic Research Council and The Finnish Research Impact Foundation.

**Giuseppe Balistreri** is a Principal Investigator at the Faculty of Medicine of University of Helsinki (Finland) and Adjunct Assistant Professor at the Queensland Brain Institute of University of Queensland (Australia). His research focuses on virus-cell interactions, from cell entry to replication and spreading, development of antivirals and vaccines. Among the viruses studied in the Balistreri’s Lab are the human pathogens SARS-CoV-2 and Respiratory Syncytial virus. The research group utilizes genetic screening approaches, high-content imaging and automated image analysis, and works on a large variety of model systems from cell lines to primary 3D-cultures of respiratory cells and neurons, as well as animal models.

**Dario Greco** is professor of bioinformatics at the Faculty of Medicine and Health Technology, Tampere University and the Director of FHAIVE, The Finnish Hub for Development and Validation of Integrated Approaches. He is also principal investigator at the Institute of Biotechnology, University of Helsinki, Finland. To date, he authored over 160 articles in peer reviewed journals in the areas of nanotoxicology, toxicogenomics, drug repositioning, network biology, data modelling and bioinformatics. He currently receives fundings from the Academy of Finland, the EU (H2020 and IMI2 programs), the Novo Nordisk Foundation, the Finnish Red Cross and the Finnish government.
