# Supplementary material for: Computationally prioritized drugs inhibit SARS-CoV-2 infection and syncytia formation
Source: Brief Bioinform. 2021 Dec 27;23(1):bbab507. doi: 10.1093/bib/bbab507 (PMC8769897; doi:10.1093/bib/bbab507)
Supplement: Supplementary_materials_bbab507 [file supplementary_materials_bbab507.pdf]

Supporting Information

Computationally prioritized drugs inhibit SARS-CoV-2 infection and syncytia formation

Angela Serra[+], Michele Fratello[+], Antonio Federico, Ravi Ojha, Riccardo Provenzani, Ervin Tasnadi, Luca Cattelani, Giusy del Giudice, Pia A. S. Kinaret, Laura A. Saarimäki, Alisa Pavel, Suvi Kuivanen, Vincenzo Cerullo, Olli Vapalahti, Peter Horvath, Antonio Di Lieto, Jari Yli-Kauhaluoma, Giuseppe Balistreri, Dario Greco[*]

[*] Corresponding Author: dario.greco@tuni.fi

[+] Equal contribution.

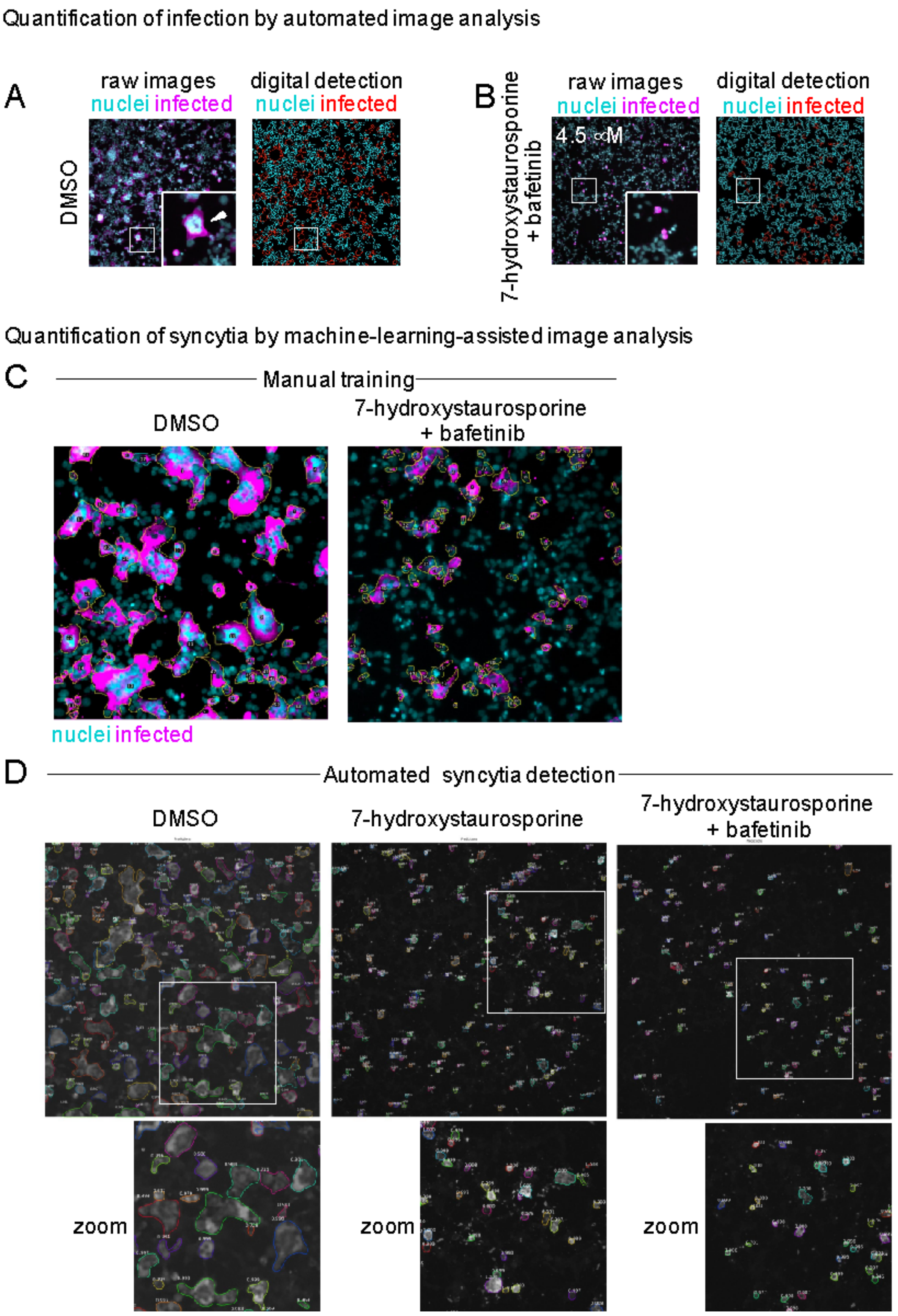

Figure S1: Image analysis A-B) Representative fluorescence images of HEK-293T-AT cells treated with indicated drugs 1 hour before infection. Cells fixed 16 hours post-infection;

blue=nuclei, magenta=infected cells. Zoomed areas from each image are indicated by white boxes; The automated identification (digital detection) of nuclei and infected cells was performed with Cell Profiler 3. C) Detection of syncytia using machine learning assisted image analysis. Images from cells treated with indicated drugs were manually annotated using the fluorescence intensities of the N protein after immunofluorescence (magenta). This annotation was used as the first training step for the machine learning analysis. D) To improve the accuracy of cell-detection, image augmentation was used to analyze the cell size and nuclear content from all images. Representative images after machine learning analysis are shown with zoomed areas indicated by white boxes.

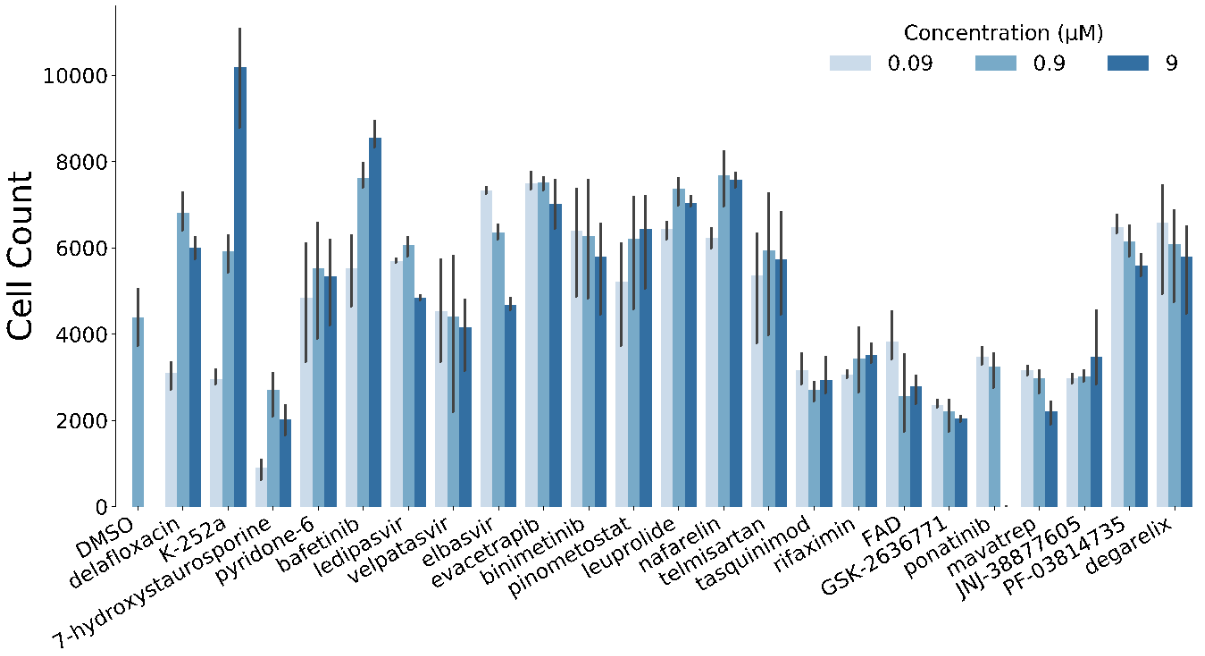

Figure S2: Number of cells counts after drug treatments. Each drug was added 45 min before infection and infected cells fixed 16 h later.

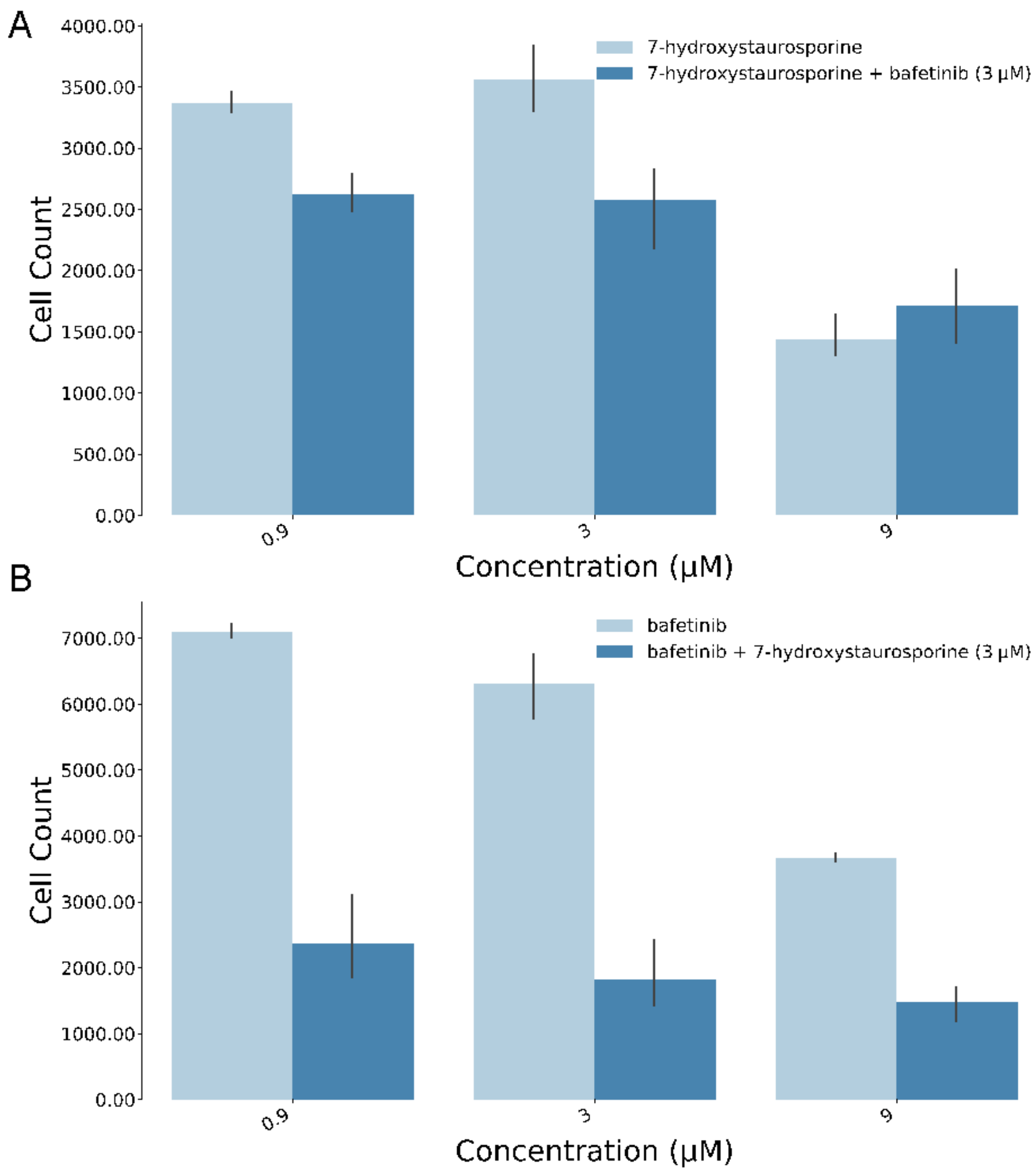

Figure S3: Cell count for the combined effect of 7-hydroxystaurosporine and bafetinib added 2 hours before infection (hbi) with 7-hydroxystaurosporine concentrations as reported and bafetinib at 3 µM (A) combined effect bafetinib and 7-hydroxystaurosporine added 2 hbi with bafetinib concentrations as shown and 7-hydroxystaurosporine at 3 µM (B).

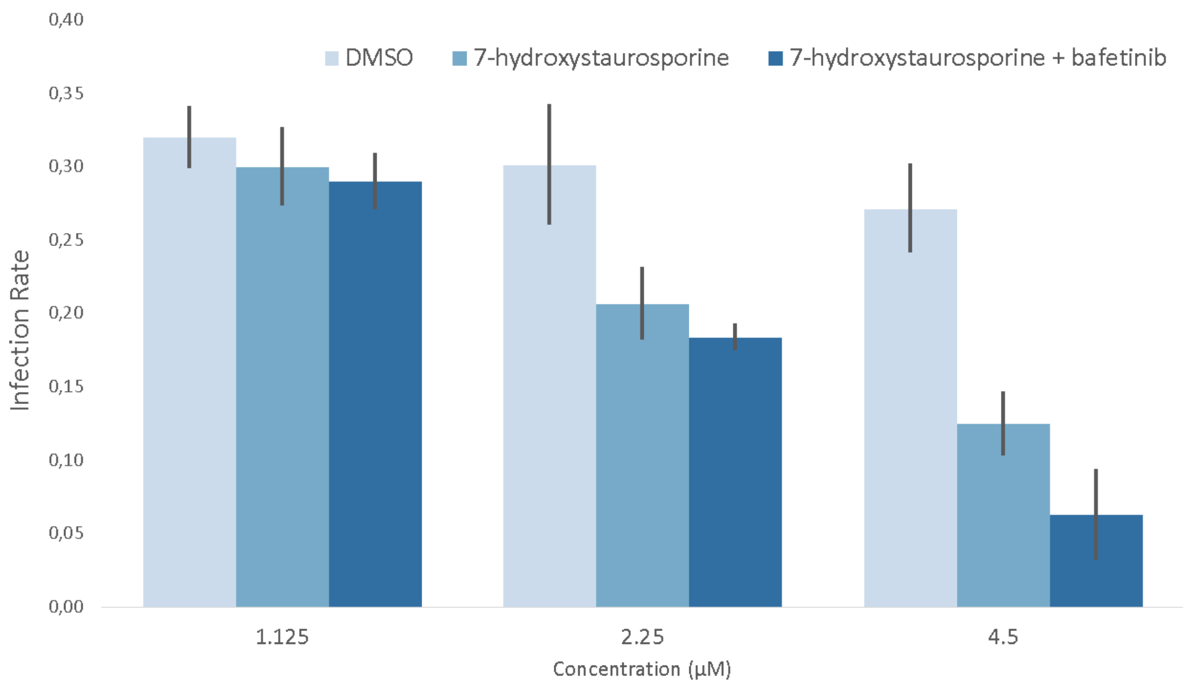

Figure S4: Combined effect of 7-hydroxystaurosporine and bafetinib added 2 hours post-infection (hpi) at indicated concentrations. Cells fixed 16 hpi.

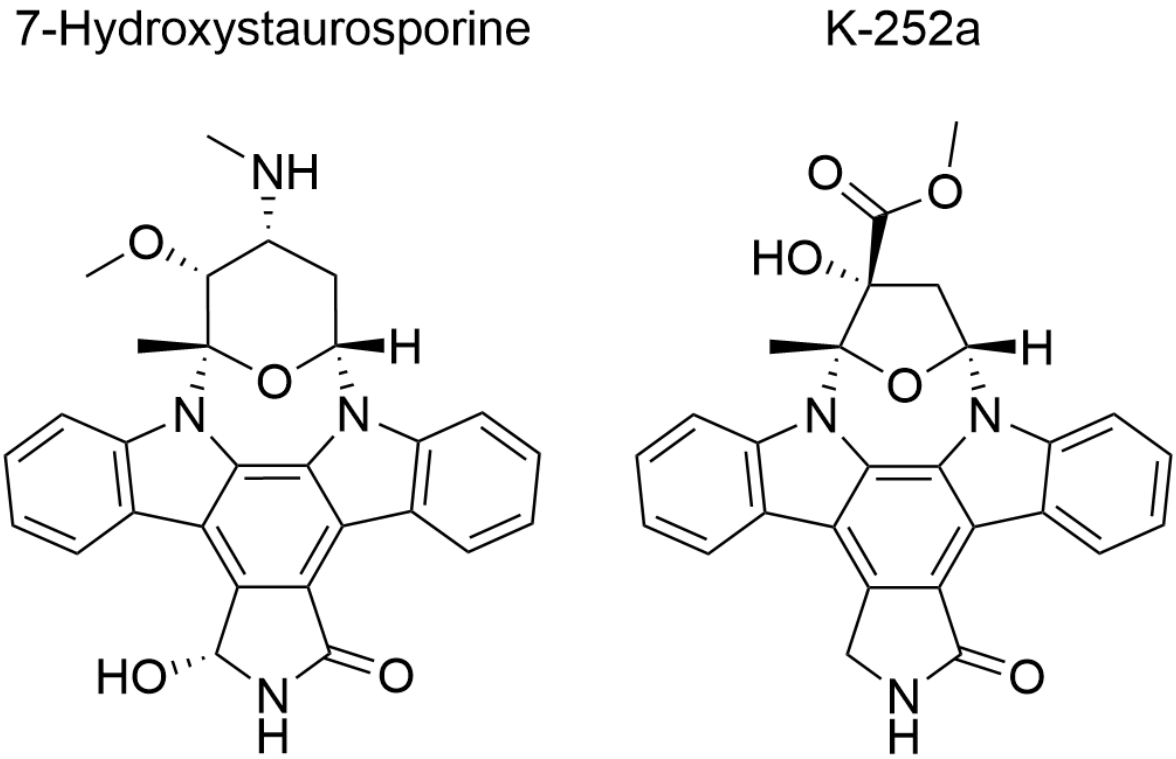

Figure S5: Structures of 7-hydroxystaurosporine and K-252a.

Supplementary Tables

Table S1: List of assays and thresholds used to define active drugs

Assay ID	Description	Activity Threshold	Nr. of Compounds	Article
CHEMBL4303805	Antiviral activity determined as inhibition of SARS-CoV-2 induced cytotoxicity of Caco-2 cells at 10 μM after 48 hours by high content imaging	> 75	5632	(Ellinger et al., 2020)
CHEMBL4303810	Antiviral activity against SARS-CoV-2 (USA-WA1/2020 strain) measured by imaging in HRCE cells at MOI 0.4 after 96 hrs (reported as hit score from 0-1 for on-disease vs off-disease activity: scores >0.6 considered hits)	> 0.6	1637	(Heiser et al., 2020)
CHEMBL4303819	Inhibition of cell viability relative to arbidol control (inhibition index > 1 indicates higher activity) measured by fluorescence (OD 590 nm) in Vero E6 cells infected with SARS-CoV-2 (strain BavPat1) at MOI 0.002 after 72 h	> 1	1520	(Touret et al., 2020)
CHEMBL4303806	Determination of IC50 values for inhibition of SARS-CoV-2 induced cytotoxicity of Caco-2 cells after 48 hours by high content imaging	< 20000	67	(Ellinger et al., 2020)
CHEMBL4303092	Overall antiviral activity against SARS-CoV-2 (isolate France/IDF0372/2020) in the Vero E6 cell line at 48 h based on three assays 1) detection of viral RNA by qRT-PCR (targeting the N-gene), 2) plaque assay using lysate 3 days after addition of compound by crystal violet staining 3) Cell viability (cytotoxicity) measuring percentage viable Vero E6 cells 48 h after compound addition by AlamarBlue assay.	Activity yes/no	59	(Gordon et al., 2020)
CHEMBL4303085	AUC (viral infection %) for SARS-CoV-2 in the Vero E6 cell	Activity yes/no	57	(Gordon et al., 2020)

	line at 48 h by immunofluorescence-based assay (detecting the viral NP protein in the nucleus of the Vero E6 cells).			
CHEMBL4303086	Cytotoxicity of compound against Vero E6 cells by MTT assay.	Activity yes/no	57	(Gordon et al., 2020)
CHEMBL4303089	AUC (cytotoxicity %) of compound against Vero E6 cells by MTT assay.	Activity yes/no	57	(Gordon et al., 2020)
CHEMBL4303091	Selectivity ratio: ratio of AUC (viral infection %) of SARS-CoV-2 in the Vero E6 cell line compared to AUC (cytotoxicity %) of compound against Vero E6 cells by MTT assay.	Activity yes/no	57	(Gordon et al., 2020)
CHEMBL4303083	IC_{50} for antiviral activity against SARS-CoV-2 in the Vero E6 cell line at 48 h by immunofluorescence-based assay (detecting the viral NP protein in the nucleus of the Vero E6 cells).	Activity yes/no	57	(Gordon et al., 2020)
CHEMBL4303811	Antiviral activity against SARS-CoV-2 (USA-WA1/2020 strain) measured by imaging in Vero cells at MOI 0.08 after 48 hrs (reported as hit score from 0-1 for on-disease vs off-disease activity: scores >0.6 considered hits)	> 0.6	32	(Heiser et al., 2020)

Table S2: Relevant chemical substructures used for DrugBank prioritization. Colors indicate if the substructure is present in bafetinib (violet), 7-hydroxystaurosporine (yellow) or both the two drugs (green).

	**Bit Substructure**
	Section 1: Hierarchic Element Counts - These bits test for the presence or count of individual chemical atoms represented by their atomic symbol.
PCFP_3	>= 16 H
PCFP_21	>= 4 O
PCFP_24	>= 1 F
PCFP_25	>= 2 F

	Section 2: Rings in a canonic Extended Smallest Set of Smallest Rings (ESSSR) ring set - These bits test for the presence or count of the described chemical ring system. An ESSSR ring is any ring which does not share three consecutive atoms with any other ring in the chemical structure. For example, naphthalene has three ESSSR rings (two phenyl fragments and the 10-membered envelope), while biphenyl will yield a count of only two ESSSR rings.
PCFP_181	>= 1 saturated or aromatic nitrogen-containing ring size 6
PCFP_186	>= 2 any ring size 6
PCFP_188	>= 2 saturated or aromatic nitrogen-containing ring size 6
PCFP_189	>= 2 saturated or aromatic heteroatom-containing ring size 6
PCFP_193	>= 3 any ring size 6
PCFP_244	>= 1 saturated or aromatic nitrogen-containing ring size 9
PCFP_260	>= 3 aromatic rings
	Section 3: Simple atom pairs - These bits test for the presence of patterns of bonded atom pairs, regardless of bond order or count.
PCFP_288	C-F
PCFP_305	N-P
PCFP_309	O-H
	Section 4: Simple atom nearest neighbors - These bits test for the presence of atom nearest neighbor patterns, regardless of bond order (denoted by ~) or count, but where bond aromaticity (denoted by :) is significant.
PCFP_364	C(~F)(~F)
PCFP_376	C(~N)(~N)
PCFP_378	C(~N)(:C)(:C)
PCFP_386	C(:C)(:C)(:C)
PCFP_392	N(~C)(~C)(~C)
PCFP_398	N(~C)(:C)(:C)
PCFP_407	O(~C)(~H)
	Section 5: Detailed atom neighborhoods - These bits test for the presence of detailed atom neighborhood patterns, regardless of count, but where bond orders are specific, bond aromaticity matches both single and double bonds, and where -, =, and # matches a single bond, double bond, and triple bond order, respectively.
PCFP_444	C(-C)(=O)

	Section 6: Simple SMARTS patterns - These bits test for the presence of simple SMARTS patterns, regardless of count, but where bond orders are specific and bond aromaticity matches both single and double bonds.
PCFP_504	Cl-C:C-[#1]
PCFP_509	S=C-N-C
PCFP_537	O=C-C-N
PCFP_561	N-C-N-C
PCFP_577	N=C-C:C-[#1]
PCFP_589	O=C-C-C=O
PCFP_594	N-C-C-C-N
PCFP_599	Cl-C:C:C-C
PCFP_601	N-C:C:C-C
PCFP_640	O-C-C-C-O
PCFP_655	N-C-N-C-C
PCFP_657	C-C-N-C-C
PCFP_668	C=C-C-O-[#1]
PCFP_675	N-C-N-C:C
PCFP_687	O=C-C-C-C-O
PCFP_697	C-C-C-C-C-C-C-C
PCFP_713	C-C(C)-C(C)-C
	Section 7: Complex SMARTS patterns - These bits test for the presence of complex SMARTS patterns, regardless of count, but where bond orders and bond aromaticity are specific.
PCFP_714	Cc1ccc(C)cc1
PCFP_717	Cc1ccc(N)cc1
PCFP_718	Cc1ccc(Cl)cc1
PCFP_729	Nc1ccc(N)cc1
PCFP_735	Cc1cc(C)ccc1
PCFP_738	Cc1cc(N)ccc1
PCFP_751	Nc1cc(Cl)ccc1
PCFP_756	Cc1c(C)cccc1

PCFP_759	Cc1c(N)cccc1
PCFP_780	CC1CCC(N)CC1
PCFP_781	CC1CCC(Cl)CC1
PCFP_792	NC1CCC(N)CC1
PCFP_814	NC1CC(Cl)CCC1
PCFP_816	ClC1CC(Cl)CCC1

Table S4: Similarity scores generated by the 3-D structural overlay of the selected structures.

Hit Structure	Overlay Structure	Overlay Similarity
7-hydroxystaurosporine	7-hydroxystaurosporine	1
7-hydroxystaurosporine	bafetinib	0.665279

Table S6: Transformations used to augment the training set.

**Transformation**	**Description**	**Parameters**	**Implementation**	**Probability (*p*)**
Non-uniform scale	Apply *scale_ratio* scaling in x-dimension and *scale_ratio* scaling in y-dimension with probability *p*.	*scale_ratio* = [0.8, 1.2]	`PIL.resize`	0.5
Flip	Flip x with probability *p*, flip y with probability *p*.	-	`numpy.{fliplr, fluipud}`	0.5
Orthogonal rotation	Rotate *degree* with probability of *p*.	*Degree*={90°, 180°, 270°}	`numpy rot{degree}`	0.5
Enhance brightness	See the Pillow documentation.[3]	*factor* =[0.8, 1.2]	`PIL.ImageEnhance.Brightness`	0.5
Enhance contrast	See the Pillow documentation.[3]	*factor*=[0.8, 1.2]	`PIL.ImageEnhance.Contrast`	0.5
Enhance sharpness	See the Pillow documentation.[3]	*factor*=[0.9, 1.1]	`PIL.ImageEnhance.Sharpness`	0.5
Add uniform	Uniform noise in the	*factor*=[0.0, 0.1]	`numpy.random`	0.5

noise	intensity range multiplied by the intensity *factor*.			
