# Supplementary material for: Computationally prioritized drugs inhibit SARS-CoV-2 infection and syncytia formation
Source: Brief Bioinform. 2021 Dec 27;23(1):bbab507. doi: 10.1093/bib/bbab507 (PMC8769897; doi:10.1093/bib/bbab507)
Supplement: Table_S3_bbab507 [file table_s3_bbab507.pdf]

Table S3: Prioritized drugs from DrugBank database.

ID	Drug ID in DrugBank database
Name	Drug Name
CID	Drug ID in PubChem database
Comp.	Number of chemical substructures in the drug, that match theones identified with the computationl approaches (bioinformatics & cheminformatics)
Active	Number of chemical substructures in the drug, that match the ones identified from previous experimental screening (from literature)
Inters.	Number of chemical substructures in the drug, that match the ones in the intersection of computational and active
ATC	Drug ATC Code code
description	Drug ATC dode description

ID	Name	CID	Comp.	Active	Inters.	ATC	description
DB06699	degarelix	16136245	45	133	34	L02BX02	endocrine therapy
DB11795	gsk-2636771	56949517	45	138	33		
DB12854	bms-908662	56931136	44	138	33		
DB01220	rifaximin	6436173	52	153	32	A07AA11	antidiarrheals, intestinal antiinflammatory/antiinfective agents
DB01220	rifaximin	6436173	52	153	32	D06AX11	antibiotics and chemotherapeutics for dermatological use
DB12095	telotristat ethyl	25025298	41	144	31		
DB11713	ruzasvir	91936863	40	147	30		
DB12559	ec-17	71587764	48	144	30		
DB05220	alisertib	24771867	43	142	30		
DB09027	ledipasvir	67505836	43	139	30		
DB08901	ponatinib	24826799	41	139	30	L01XE24	antineoplastic agents
**DB11851**	**bafetinib**	**11387605**	**36**	**129**	**30**		
DB07969	3-[3-(4-methylpiperazin-1-yl)-7-(trifluoromethyl)quinoxalin-5-yl]phenol	25113181	37	128	30		
DB01259	lapatinib	208908	38	147	29	L01XE07	antineoplastic agents
DB05772	rabeximod	56841552	37	140	29		
DB05424	canertinib	156414	35	138	29		
DB11963	dacomitinib	11511120	35	138	29		
DB06925	3-(2-aminoquinazolin-6-yl)-4-methyl-n-[3-(trifluoromethyl)phenyl]benzamide	15991573	34	131	29		
DB05412	talmapimod	9871074	39	130	29		
DB12817	zoliflodacin	76685216	49	113	29		

DB02873	1-(2,6-dichlorophenyl)-5-(2,4-difluorophenyl)-7-piperazin-1-yl-3,4-dihydroquinazolin-2(1h)-one	4630909	36	97	29		
DB12691	uk-432097	9833519	38	145	28		
DB11191	cobamamide	70678541	46	139	28	B03BA04	antianemic preparations
DB00666	nafarelin	25077405	44	137	28	H01CA02	pituitary and hypothalamic hormones and analogues
DB13061	mln8054	11712649	37	137	28		
DB00966	telmisartan	65999	37	136	28	C09CA07	agents acting on the renin-angiotensin system
DB13878	pibrentasvir	58031952	37	136	28		
DB08916	afatinib	10184653	36	136	28	L01XE13	antineoplastic agents
DB13060	cep-37440	71721648	41	133	28		
DB12352	bizelesin	60794	41	132	28		
DB05168	vintafolide	122173811	56	131	28	L01CA06	antineoplastic agents
DB06785	ganirelix	16130957	39	130	28	H01CC01	pituitary and hypothalamic hormones and analogues
DB00050	cetrorelix	25074887	39	127	28	H01CC02	pituitary and hypothalamic hormones and analogues
DB12581	ozarelix	25080293	39	127	28		
**DB01933**	**7-hydroxystaurosporine**	**72271**	**46**	**126**	**28**		
DB12868	intoplicine	65954	38	124	28		
DB12424	mk-3207	25019940	41	123	28		
DB12167	ly-3023414	57519748	36	121	28		
DB12073	albaconazole	208952	38	116	28		
DB11853	relugolix	10348973	40	115	28		
DB11581	venetoclax	49846579	47	146	27		
DB11613	velpatasvir	67683363	39	146	27		

DB12269	pf-06273340	66571548	35	144	27		
DB06636	isavuconazonium	6918606	34	144	27		
DB02349	nicotinamide-adenine-dinucleotide-5-hydroxy-4-oxonorvaline	5289029	48	142	27		
DB11987	gsk-2018682	24988201	45	141	27		
DB07326	6-chloro-n-pyrimidin-5-yl-3-{[3-(trifluoromethyl)phenyl]amino}-1,2-benzisoxazole-7-carboxamide	23658582	44	141	27		
DB12037	vedroprevir	25167947	44	140	27		
DB12876	gs-9256	24823649	47	139	27		
DB11913	ly-2090314	10029385	38	139	27		
DB11689	selumetinib	10127622	49	138	27		
DB13005	rebastinib	25066467	38	136	27		
DB12183	sapitinib	11488320	34	134	27		
DB11904	flumatinib	46848036	30	133	27		
DB12674	lurbinectedin	57327016	42	132	27		
DB12218	capivasertib	25227436	34	131	27		
DB02332	flavin-n7 protonated-adenine dinucleotide	131704201	41	130	27		
DB03147	flavin adenine dinucleotide	643975	41	130	27		
DB03482	8-demethyl-8-dimethylamino-flavin-adenine-dinucleotide	5289282	41	130	27		
DB12756	tak-901	16124208	40	130	27		
DB08351	n-cyclopropyl-4-methyl-3-{2-[(2-morpholin-4-ylethyl)amino]quinazolin-6-yl}benzamide	24963046	33	130	27		

DB11852	tegobuvir	23649154	33	129	27		
DB12640	cp-609754	46208720	37	127	27		
DB02152	k-252a	3035817	43	126	27		
DB11906	acyline	16137348	35	125	27		
DB04882	edotecarin	9808998	51	121	27		
DB11943	delafloxacin	487101	41	121	27		
DB08166	(4r)-7-chloro-9-methyl-1-oxo-1,2,4,9-tetrahydrospiro[beta-carboline-3,4'-piperidine]-4-carbonitrile	24851689	34	119	27		
DB13059	pf-03814735	51346455	34	119	27		
DB07242	(4r)-7,8-dichloro-1',9-dimethyl-1-oxo-1,2,4,9-tetrahydrospiro[beta-carboline-3,4'-piperidine]-4-carbonitrile	24851688	37	116	27		
DB12185	exatecan	151115	39	115	27		
DB12875	mavatrep	17751090	34	115	27		
DB00246	ziprasidone	60854	33	113	27	N05AE04	psycholeptics
DB00246	ziprasidone	60854	33	113	27	N05AE04	psycholeptics
DB11574	elbasvir	71661251	36	141	26		
DB01831	tryptophanyl-5'amp	446202	40	139	26		
DB03331	n-naphthalen-1-ylmethyl-2'-[3,5-dimethoxybenzamido]-2'-deoxy-adenosine	446186	38	137	26		
DB11791	capmatinib	25145656	34	137	26		
DB06788	histrelin	56927879	40	136	26	L02AE05	endocrine therapy

DB04673	4-[(5-chloroindol-2-yl)sulfonyl]-2-(2-methylpropyl)-1-[[5-(pyridin-4-yl)pyrimidin-2-yl]carbonyl]piperazine	49866889	32	136	26		
DB01777	coa-s-trimethylene-acetyl-tryptamine	446814	40	135	26		
DB12114	poziotinib	25127713	32	135	26		
DB03341	coa-s-acetyl 5-bromotryptamine	446811	49	134	26		
DB02931	coa-s-acetyl tryptamine	444837	40	134	26		
DB09265	lixisenatide	131704317	38	133	26	A10BX10	drugs used in diabetes
DB08881	vemurafenib	42611257	35	133	26	L01XE15	antineoplastic agents
DB12121	entospletinib	59473233	35	133	26		
DB06834	n-(2-hydroxy-1,1-dimethylethyl)-1-methyl-3-(1h-pyrrolo[2,3-b]pyridin-2-yl)-1h-indole-5-carboxamide	9968957	34	132	26		
DB12001	abemaciclib	46220502	30	132	26		
DB13113	jnj-38877605	46911863	32	131	26		
DB11966	bms-986094	46700744	43	129	26		
DB12011	fevipiprant	23582412	36	129	26		
DB12323	radotinib	16063245	31	129	26		
DB11967	binimetinib	10288191	46	128	26		
DB04868	nilotinib	644241	31	128	26	L01XE08	antineoplastic agents
DB00115	cyanocobalamin	70678590	44	127	26	B03BA01	antianemic preparations
DB00115	cyanocobalamin	70678590	44	127	26	B03BA01	antianemic preparations
DB00115	cyanocobalamin	70678590	44	127	26	B03BA01	antianemic preparations

DB07528	3-(2-aminoquinazolin-6-yl)-4-methyl-1-[3-(trifluoromethyl)phenyl]pyridin-2(1h)-one	24812719	29	127	26		
DB00200	hydroxocobalamin	70678542	44	126	26	B03BA03	antianemic preparations
DB00200	hydroxocobalamin	70678542	44	126	26	V03AB33	all other therapeutic products
DB03614	methylcobalamin	71306319	44	126	26		
DB00320	dihydroergotamine	10531	37	126	26	N02CA01	analgesics
DB00320	dihydroergotamine	10531	37	126	26	N02CA01	analgesics
DB00320	dihydroergotamine	10531	37	126	26	N02CA01	analgesics
DB01346	quinidine barbiturate	53461739	34	126	26		
DB07261	thieno[3,2-b]pyridine-2-sulfonic acid [1-(1-amino-isoquinolin-7-ylmethyl)-2-oxo-pyrroldin-3-yl]-amide	445480	34	125	26		
DB00106	abarelix	16131215	39	123	26	L02BX01	endocrine therapy
DB04716	2-(1,1-dimethylethyl)9-fluoro-3,6-dihydro-7h-benz[h]-imidaz[4,5-f]isoquinolin-7-one	5494425	38	122	26		
DB12981	xl-888	60147023	40	117	26		
DB09335	alatrofloxacin	3086677	37	117	26		
DB11986	entrectinib	25141092	41	116	26		
DB12211	psi-697	12004316	38	116	26		
DB07006	9-hydroxy-6-(3-hydroxypropyl)-4-(2-methoxyphenyl)pyrrolo[3,4-c]carbazole-1,3(2h,6h)-dione	10364585	42	114	26		

DB07265	3-(9-hydroxy-1,3-dioxo-4-phenyl-2,3-dihydropyrrolo[3,4-c]carbazol-6(1h)-yl)propanoic acid	9978312	41	114	26		
DB04330	bilh 434	5287508	49	113	26		
DB07612	6-(3-aminophenyl)-n-(tert-butyl)-2-(trifluoromethyl)quinazolin-4-amine	5288016	30	113	26		
DB03865	6-chloro-2-(2-hydroxy-biphenyl-3-yl)-1h-indole-5-carboxamidine	445843	34	109	26		
DB02132	zenarestat	5724	40	98	26		
DB02733	purvalanol	448991	34	142	25		
DB07786	1-((1r,2s)-1-{2-[2-(4-chlorophenyl)-1,3-benzoxazol-7-yl]ethyl}-2-hydroxypropyl)-1h-imidazole-4-carboxamide	46937091	38	141	25		
DB12760	gw-493838	9810927	38	140	25		
DB04591	n-{2,2-difluoro-2-[(2r)-piperidin-2-yl]ethyl}-2-[2-(1h-1,2,4-triazol-1-yl)benzyl][1,3]oxazolo[4,5-c]pyridin-4-amine	5287492	39	138	25		
DB12318	bms-599626	10437018	33	137	25		
DB01284	tetracosactide	16133751	41	136	25	H01AA02	pituitary and hypothalamic hormones and analogues
DB03168	nicotinamide adenine dinucleotide cyclohexanone	131704230	40	136	25		

DB12669	4sc-203	44467821	36	136	25		
DB04071	cpad	444170	38	135	25		
DB12848	pf-04217903	17754438	31	135	25		
DB04931	afamelanotide	16197727	41	134	25	D02BB02	emollients and protectives
DB05479	czen 002	16129664	41	134	25		
DB08402	2-[(2,4-dichlorobenzoyl)amino]-5-(pyrimidin-2-yloxy)benzoic acid	5289162	32	133	25		
DB13919	candesartan	2541	40	132	25	C09CA06	agents acting on the renin-angiotensin system
DB06825	triptorelin	25074470	39	132	25	L02AE04	endocrine therapy
DB06825	triptorelin	25074470	39	132	25	L02AE04	endocrine therapy
DB00317	gefitinib	123631	30	131	25	L01XE02	antineoplastic agents
DB13041	odalasvir	71474517	34	129	25		
DB00705	delavirdine	5625	33	125	25	J05AG02	antivirals for systemic use
DB08221	n-{4-methyl-3-[(3-pyrimidin-4-ylpyridin-2-yl)amino]phenyl}-3-(trifluoromethyl)benzamide	16040281	28	125	25		
DB08512	6-amino-2-[(1-naphthylmethyl)amino]-3,7-dihydro-8h-imidazo[4,5-g]quinazolin-8-one	16750097	38	124	25		
DB11743	ipatasertib	24788740	36	124	25		
DB12721	ro-5028442	59657596	35	124	25		
DB04960	tipifarnib	159324	31	124	25		
DB08203	7-[2-methoxy-1-(methoxymethyl)ethyl]-7h-pyrrolo[3,2-f] quinazoline-1,3-diamine	447099	31	124	25		

DB11651	dactolisib	11977753	30	124	25		
DB05403	cep-1347	133005	41	123	25		
DB11975	tak-448	46700761	46	122	25		
DB12985	quisinostat	11538455	34	122	25		
DB03082	6-[(z)-amino(imino)methyl]-n-[4-(aminomethyl)phenyl]-4-(pyrimidin-2-ylamino)-2-naphthamide	448605	33	122	25		
DB09143	sonidegib	24775005	31	122	25	L01XX48	antineoplastic agents
DB12228	telcagepant	11319053	32	120	25		
DB00541	vincristine	5978	40	118	25	L01CA02	antineoplastic agents
DB00570	vinblastine	13342	40	118	25	L01CA01	antineoplastic agents
DB12897	mk-7622	46207733	34	118	25		
DB08365	8-bromo-4-(2-chlorophenyl)-n-(2-hydroxyethyl)-6-methyl-1,3-dioxo-1,2,3,6-tetrahydropyrrolo[3,4-e]indole-7-carboxamide	11691442	45	117	25		
DB12524	bi-671800	45270144	27	117	25		
DB11363	alectinib	49806720	38	116	25		
DB12924	ozenoxacin	9863827	39	115	25		
DB12562	setipiprant	49843471	31	115	25		
DB00471	montelukast	5281040	31	114	25	R03DC03	drugs for obstructive airway diseases
DB11712	tezacaftor	46199646	36	112	25		
DB12140	dilmapimod	10297982	28	112	25		
DB03933	c-1027 aromatized chromophore	5289301	44	109	25		
DB12325	idasanutlin	53358942	36	105	25		
DB12030	fluorescein lisicol	76958588	44	100	25		
DB12340	navitoclax	24978538	30	97	25		

DB01948	1-(2,6-dichlorophenyl)-5-(2,4-difluorophenyl)-7-piperidin-4-yl-3,4-dihydroquinolin-2(1h)-one	447725	34	95	25		
DB02794	(2s)-2-({4-[(2s)-1-(2-amino-4-oxo-1,4-dihydro-6-quinazolinyl)-3-{[2-({(2r,3r,4s,5r)-3,4-dihydroxy-5-[(phosphonooxy)methyl]tetrahydro-2-furanyl}amino)-2-oxoethyl]amino}-2-hydroxy-2-propanyl]benzoyl}amino)pentanedioic acid	446514	46	94	25		
DB07827	4-{[1-methyl-2,4-dioxo-6-(3-phenylprop-1-yn-1-yl)-1,4-dihydroquinazolin-3(2h)-yl]methyl}benzoic acid	10224181	36	92	25		
DB12934	granotapide	11607299	34	88	25		
DB11984	letaxaban	11641515	35	71	25		
DB05075	tg-100801	11973736	36	145	24		
DB12138	pf-03715455	11714580	33	138	24		
DB11796	fostemsavir	11319217	45	137	24		
DB02820	1-azepan-1-yl-2-phenyl-2-(4-thioxo-1,4-dihydro-pyrazolo[3,4-d]pyrimidin-5-yl)ethanone adduct	131704220	41	137	24		
DB12799	laniquidar	6450806	35	137	24		
DB12706	seletalisib	56928390	34	137	24		
DB13088	azd-0424	9893171	33	137	24		

DB05944	varlitinib	42642648	30	137	24		
DB09034	suvorexant	24965990	42	136	24		
DB12331	labetuzumab govitecan	91668184	40	136	24		
DB03020	5-beta-d-ribofuranosylnicotinamide adenine dinucleotide	444215	42	134	24		
DB04099	deamido-nad+	165491	40	134	24		
DB04477	n-1,2,3,4-tetrahydronaphth-1-yl-2'-[3,5-dimethoxybenzamido]-2'-deoxy-adenosine	5289484	37	134	24		
DB08057	n-(2-chloro-6-methylphenyl)-8-[(3s)-3-methylpiperazin-1-yl]imidazo[1,5-a]quinoxalin-4-amine	46937119	33	134	24		
DB11973	tesevatinib	10458325	33	133	24		
DB00014	goserelin	5311128	45	131	24	L02AE03	endocrine therapy
DB08707	4-[3-(4-chlorophenyl)-2,1-benzisoxazol-5-yl]pyrimidin-2-amine	2765355	40	131	24		
DB13002	hki-357	11238512	30	131	24		
DB02323	em-1745	446201	38	130	24		
DB12690	ly-2584702	25118925	33	130	24		
DB07783	1-((1r)-1-(hydroxymethyl)-3-{6-[(3-phenylpropanoyl)amino]-1h-indol-1-yl}propyl)-1h-imidazole-4-carboxamide	448903	31	130	24		
DB11828	neratinib	9915743	30	130	24		
DB00007	leuprolide	657181	38	129	24		

DB00644	gonadorelin	36523	38	129	24	H01CA01	pituitary and hypothalamic hormones and analogues
DB00644	gonadorelin	36523	38	129	24	V04CM01	diagnostic agents
DB11832	crenolanib	10366136	35	129	24		
DB06876	n-{5-[4-(4-methylpiperazin-1-yl)phenyl]-1h-pyrrolo[2,3-b]pyridin-3-yl}nicotinamide	6914568	32	129	24		
DB12920	pinometostat	57345410	32	129	24		
DB09330	osimertinib	71496458	31	129	24		
DB08339	6-(2,6-dichlorophenyl)-2-{[3-(hydroxymethyl)phenyl]amino}-8-methylpyrido[2,3-d]pyrimidin-7(8h)-one	447700	28	129	24		
DB08449	2-(3-((4,5,7-trifluorobenzo[d]thiazol-2-yl)methyl)-1h-pyrrolo[2,3-b]pyridin-1-yl)acetic acid	10150441	26	129	24		
DB11663	pictilisib	17755052	35	128	24		
DB01674	[2-(1-amino-2-hydroxy-propyl)-4-(4-fluoro-1h-indol-3-ylmethyl)-5-hydroxy-imidazol-1-yl]-acetic acid	17753789	32	128	24		
DB13040	gandotinib	46213929	31	128	24		
DB12978	pexidartinib	25151352	28	128	24		
DB08882	linagliptin	10096344	35	127	24	A10BH05	drugs used in diabetes
DB07514	3-(2-aminoquinazolin-6-yl)-1-(3,3-dimethylindolin-6-yl)-4-methylpyridin-2(1h)-one	24812717	30	127	24		

DB04057	beta-dadf, msa, multisubstrate adduct inhibitor	6323417	42	126	24		
DB04727	1-{4-[4-amino-6-(4-methoxyphenyl)furo[2,3-d]pyrimidin-5-yl]phenyl}-3-[2-fluoro-5-(trifluoromethyl)phenyl]urea	5326956	37	126	24		
DB13095	jtk-853	57519700	34	126	24		
DB07000	n-{2,4-difluoro-3-[(5-pyridin-3-yl-1h-pyrrolo[2,3-b]pyridin-3-yl)carbonyl]phenyl}ethanesulfonamide	24180720	33	126	24		
DB02830	fr236913	447546	32	126	24		
DB12158	filorexant	25128145	32	126	24		
DB02084	cra_17312	131704188	31	126	24		
DB12774	azd-8055	25262965	30	126	24		
DB07856	6-{4-[4-(4-chlorophenyl)piperidin-4-yl]phenyl}-9h-purine	16122633	29	126	24		
DB11740	mk-1775	24856436	34	125	24		
DB01691	indole naphthyridinone	5288607	28	125	24		
DB04852	implitapide	5745206	31	124	24		
DB01347	saprisartan	60921	44	123	24		
DB06595	midostaurin	9829523	40	123	24		
DB12550	taladegib	49848070	30	123	24		

DB08148	1-[4-(4-chlorophenyl)-1-(7h-pyrrolo[2,3-d]pyrimidin-4-yl)piperidin-4-yl]methanamine	24762195	29	123	24		
DB07053	2-{5-[3-(7-propyl-3-trifluoromethylbenzo[d]isoxazol-6-yloxy)propoxy]indol-1-yl}ethanoic acid	9547900	44	122	24		
DB02868	3"-(beta-chloroethyl)-2",4"-dioxo-3, 5"-spiro-oxazolidino-4-deacetoxy-vinblastine	157684	37	122	24		
DB00696	ergotamine	8223	34	122	24	N02CA02	analgesics
DB00696	ergotamine	8223	34	122	24	N02CA02	analgesics
DB00696	ergotamine	8223	34	122	24	N02CA02	analgesics
DB00696	ergotamine	8223	34	122	24	N02CA02	analgesics
DB00696	ergotamine	8223	34	122	24	N02CA02	analgesics
DB07943	2-{4-[5-(4-chlorophenyl)-4-pyrimidin-4-yl-1h-pyrazol-3-yl]piperidin-1-yl}-2-oxoethanol	9865587	30	122	24		
DB04689	2-{5-[3-(6-benzoyl-1-propylnaphthalen-2-yloxy)propoxy]indol-1-yl}ethanoic acid	6102812	33	121	24		
DB07862	7-(1-ethyl-propyl)-7h-pyrrolo-[3,2-f]quinazoline-1,3-diamine	1881	30	121	24		
DB04869	olcegepant	6918509	39	119	24		

DB07406	(4r)-n-[4-({[2-(dimethylamino)ethyl]amino}carbonyl)-1,3-thiazol-2-yl]-4-methyl-1-oxo-2,3,4,9-tetrahydro-1h-beta-carboline-6-carboxamide	16741237	31	119	24		
DB11694	ilorasertib	46207586	29	119	24		
DB00361	vinorelbine	44424639	39	118	24	L01CA04	antineoplastic agents
DB08730	3-fluoro-5-morpholin-4-yl-n-[1-(2-pyridin-4-ylethyl)-1h-indol-6-yl]benzamide	5326869	30	118	24		
DB11907	rociletinib	57335384	28	118	24		
DB00309	vindesine	40839	39	117	24	L01CA03	antineoplastic agents
DB01157	trimetrexate	5583	34	117	24	P01AX07	antiprotozoals
DB09048	netupitant	6451149	27	117	24		
DB12586	anhydrovinblastine	11104750	39	116	24		
DB08703	12-(2-hydroxyethyl)-2-(1-methylethoxy)-13,14-dihydronaphtho[2,1-a]pyrrolo[3,4-c]carbazol-5(12h)-one	25203955	35	115	24		
DB09280	lumacaftor	16678941	35	115	24		
DB12306	cipargamin	44469321	34	115	24		
DB13104	x-396	56960447	33	115	24		
DB12960	incb-9471	49871007	38	114	24		
DB07570	3-cyclohexyl-1-(2-morpholin-4-yl-2-oxoethyl)-2-phenyl-1h-indole-6-carboxylic acid	4369534	32	114	24		
DB00613	amodiaquine	2165	29	114	24	P01BA06	antiprotozoals
DB01771	cra_10991	447488	34	111	24		

DB01263	posaconazole	468595	33	111	24	J02AC04	antimycotics for systemic use
DB01200	bromocriptine	31101	41	110	24	G02CB01	other gynecologicals
DB01200	bromocriptine	31101	41	110	24	G02CB01	other gynecologicals
DB01200	bromocriptine	31101	41	110	24	N04BC01	anti-parkinson drugs
DB07220	n-[5-(1,1-dioxidoisothiazolidin-2-yl)-1h-indazol-3-yl]-2-(4-piperidin-1-ylphenyl)acetamide	10138993	33	109	24		
DB12047	ganetespib	23624255	37	108	24		
DB12253	gdc-0810	56941241	35	108	24		
DB12778	rivipansel	44232546	46	98	24		
DB12526	batefenterol	10372836	41	98	24		
DB11611	lifitegrast	11965427	39	98	24		
DB11655	evacetrapib	49836058	32	95	24		
DB12051	setrobuvir	126843190	40	91	24		
DB01137	levofloxacin	149096	35	91	24	J01MA12	antibacterials for systemic use
DB01137	levofloxacin	149096	35	91	24	J01MA12	antibacterials for systemic use
DB01137	levofloxacin	149096	35	91	24	S01AE05	ophthalmologicals
DB01165	ofloxacin	4583	35	91	24	J01MA01	antibacterials for systemic use
DB01165	ofloxacin	4583	35	91	24	J01MA01	antibacterials for systemic use
DB01165	ofloxacin	4583	35	91	24	S01AE01	ophthalmologicals
DB01165	ofloxacin	4583	35	91	24	S02AA16	otologicals
DB03034	d-levofloxacin	452723	35	91	24		
DB12447	nadifloxacin	4410	36	87	24	D10AF05	anti-acne preparations
DB12329	eravacycline	54726192	39	86	24		
DB12454	zalypsis	16061448	36	86	24		

DB06985	2-[({4-[2-(trifluoromethyl)phenyl]piperidin-1-yl}carbonyl)amino]benzoic acid	25138295	31	86	24		
DB00560	tigecycline	54686904	41	85	24	J01AA12	antibacterials for systemic use
DB12949	pf-03382792	71451950	31	83	24		
DB12393	fanapanel	208953	31	79	24		
DB01218	halofantrine	37393	32	68	24	P01BX01	antiprotozoals
DB06938	4-[[2-[[4-chloro-3-(trifluoromethyl)phenyl]amino]-3h-benzimidazol-5-yl]oxy]-n-methyl-pyridine-2-carboxamide	11167118	33	141	23		
DB12165	presatovir	58029842	35	138	23		
DB12154	itacitinib	53380437	33	137	23		
DB00563	methotrexate	126941	30	137	23	L01BA01	antineoplastic agents
DB00563	methotrexate	126941	30	137	23	L04AX03	immunosuppressants
DB12660	samatasvir	58310140	29	136	23		
DB11745	otenabant	10052040	28	135	23		
DB00796	candesartan cilexetil	2540	39	134	23		
DB04421	nicotinamide adenine dinucleotide 3-pentanone adduct	131704281	38	134	23		

DB02732	[(2r,3s,4r,5r)-5-(4-acetonyl-3-carbamoyl-pyridin-1-ium-1-yl)-3,4-dihydroxy-tetrahydrofuran-2-yl]methyl [[(2r,3s,4r,5r)-5-(6-aminopurin-9-yl)-3,4-dihydroxy-tetrahydrofuran-2-yl]methoxy-hydroxy-phosphoryl] phosphate	131704217	38	132	23		
DB06908	(2s)-3-(1-{[2-(2-chlorophenyl)-5-methyl-1,3-oxazol-4-yl]methyl}-1h-indol-5-yl)-2-ethoxypropanoic acid	9549225	33	132	23		
DB02498	carba-nicotinamide-adenine-dinucleotide	163884	39	131	23		
DB03363	3-acetylpyridine adenine dinucleotide	131704240	37	131	23		
DB07546	[1-(6-{6-[(1-methylethyl)amino]-1h-indazol-1-yl}pyrazin-2-yl)-1h-pyrrol-3-yl]acetic acid	16224058	33	131	23		
DB07845	2-fluoro-6-{[2-({2-methoxy-4-[(methylsulfonyl)methyl]phenyl}amino)-7h-pyrrolo[2,3-d]pyrimidin-4-yl]amino}benzamide	25113171	33	131	23		
DB07337	4-[4-amino-6-(5-chloro-1h-indol-4-ylmethyl)-[1,3,5]triazin-2-ylamino]-benzonitrile	448533	31	131	23		

DB03907	n-{3-[5-(6-amino-purin-9-yl)-3,4-dihydroxy-tetrahydro-furan-2-yl]-allyl}-2,3-dihydroxy-5-nitro-benzamide	4369234	40	130	23		
DB03962	nicotinamide 8-bromo-adenine dinucleotide phosphate	131704263	39	130	23		
DB02483	etheno-nad	131675359	37	130	23		
DB03461	nicotinamide adenine dinucleotide phosphate	57525501	37	130	23		
DB09272	eluxadoline	11250029	37	130	23		
DB04885	cilansetron	6918107	32	130	23	A03AE03	drugs for functional gastrointestinal disorders
DB12369	sotrastaurin	10296883	31	130	23		
DB13138	neladenoson bialanate	56848985	28	130	23		
DB12966	falnidamol	6918508	25	130	23		
DB12742	amuvatinib	11282283	34	129	23		
DB04867	lintitript	122077	28	129	23		
DB08054	1-(1-methylethyl)-3-quinolin-6-yl-1h-pyrazolo[3,4-d]pyrimidin-4-amine	24905152	30	128	23		
DB12557	fk-614	9869229	30	128	23		
DB12302	cp-724714	9874913	29	128	23		
DB08822	azilsartan medoxomil	11238823	36	127	23	C09CA09	agents acting on the renin-angiotensin system
DB05868	ciluprevir	9853710	35	127	23		

DB06969	2-amino-4-[2,4-dichloro-5-(2-pyrrolidin-1-ylethoxy)phenyl]-n-ethylthieno[2,3-d]pyrimidine-6-carboxamide	25210273	34	127	23		
DB07239	7-(aminomethyl)-6-(2-chlorophenyl)-1-methyl-1h-benzimidazole-5-carbonitrile	11572962	30	127	23		
DB09075	edoxaban	10280735	30	127	23		
DB09078	lenvatinib	9823820	39	126	23	L01XE29	antineoplastic agents
DB01254	dasatinib	3062316	32	126	23	L01XE06	antineoplastic agents
DB01419	antrafenine	68723	30	126	23		
DB11808	faldaprevir	42601552	45	125	23	J05AE13	antivirals for systemic use
DB04117	4-(n,n-dimethylamino)cinnamoyl-coa	445461	36	125	23		
DB08463	(2r)-2-({9-(1-methylethyl)-6-[(4-pyridin-2-ylbenzyl)amino]-9h-purin-2-yl}amino)butan-1-ol	10224714	27	125	23		
DB08116	(3r)-4-{[(3,4-dihydroxyphenyl)acetyl]oxy}-n-(2-formylindolizin-3-yl)-3-sulfino-d-valine	46937126	36	124	23		
DB08268	6-amino-4-[2-(4-methylphenyl)ethyl]-1,7-dihydro-8h-imidazo[4,5-g]quinazolin-8-one	5326929	35	124	23		
DB12010	fostamatinib	11671467	34	124	23		
DB13156	inosine pranobex	131704326	34	124	23	J05AX05	antivirals for systemic use

DB04903	pagoclone	131664	30	124	23		
DB08005	4-{[5-chloro-4-(1h-indol-3-yl)pyrimidin-2-yl]amino}-n-ethylpiperidine-1-carboxamide	16129582	28	124	23		
DB12282	defactinib	25117126	26	124	23		
DB03916	4-{2-[4-(2-aminoethyl)piperazin-1-yl]pyridin-4-yl}-n-(3-chloro-4-methylphenyl)pyrimidin-2-amine	656967	24	124	23		
DB08674	(20s)-19,20,21,22-tetrahydro-19-oxo-5h-18,20-ethano-12,14-etheno-6,10-metheno-18h-benz[d]imidazo[4,3-k][1,6,9,12]oxatriaza-cyclooctadecosine-9-carbonitrile	23646577	32	123	23		
DB12457	rimegepant	51049968	32	123	23		
DB08150	4-(4-chlorobenzyl)-1-(7h-pyrrolo[2,3-d]pyrimidin-4-yl)piperidin-4-aminium	42627562	28	123	23		
DB07113	(2s)-6-(2,4-diamino-6-ethylpyrimidin-5-yl)-2-(3,5-difluorophenyl)-4-(3-methoxypropyl)-2h-1,4-benzoxazin-3(4h)-one	20843156	27	123	23		
DB04877	voacamine	11953931	38	122	23		
DB09063	ceritinib	57379345	29	122	23	L01XE28	antineoplastic agents
DB01026	ketoconazole	3823	28	122	23	D01AC08	antifungals for dermatological use

DB01026	ketoconazole	3823	28	122	23	G01AF11	gynecological antiinfectives and antiseptics
DB01026	ketoconazole	3823	28	122	23	J02AB02	antimycotics for systemic use
DB08149	1-[4-(4-chlorobenzyl)-1-(7h-pyrrolo[2,3-d]pyrimidin-4-yl)piperidin-4-yl]methanamine	11696113	28	122	23		
DB07021	(7r,8r)-8-(2,4,5-trifluorophenyl)-6,7,8,9-tetrahydroimidazo[1,2-a:4,5-c']dipyridin-7-amine	15953860	27	122	23		
DB07602	s-{3-[(4-anilinoquinazolin-6-yl)amino]-3-oxopropyl}-l-cysteine	46937083	27	122	23		
DB12443	sonedenoson	9910098	27	122	23		
DB07257	4-(2-chlorophenyl)-8-(2-hydroxyethyl)-6-methylpyrrolo[3,4-e]indole-1,3(2h,6h)-dione	11602832	34	121	23		
DB08976	floctafenine	76958517	31	121	23	N02BG04	analgesics
DB12522	toreforant	23650961	30	121	23		
DB07563	1-{7-cyclohexyl-6-[4-(4-methylpiperazin-1-yl)benzyl]-7h-pyrrolo[2,3-d]pyrimidin-2-yl}methanamine	46937081	27	121	23		
DB02010	staurosporine	44259	39	120	23		
DB02026	furo[2,3d]pyrimidine antifolate	158376	38	120	23		
DB03621	l-709,587	5496872	35	120	23		

DB06932	10,11-dimethoxy-4-methyldibenzo[c,f]-2,7-naphthyridine-3,6-diamine	17754396	29	120	23		
DB12566	decernotinib	59422203	28	120	23		
DB04437	cysteine-methylene-carbamoyl-1,10-phenanthroline	17754108	27	120	23		
DB09295	talniflumate	48229	34	119	23		
DB07994	n~3~-[5-(1h-indol-6-yl)-2-(pyridin-2-ylmethoxy)benzyl]pyridine-2,3-diamine	11633167	31	119	23		
DB01761	4-[5-[2-(1-phenyl-ethylamino)-pyrimidin-4-yl]-1-methyl-4-(3-trifluoromethylphenyl)-1h-imidazol-2-yl]-piperidine	447721	27	119	23		
DB07564	6-amino-2-[(2-morpholin-4-ylethyl)amino]-3,7-dihydro-8h-imidazo[4,5-g]quinazolin-8-one	23643565	34	118	23		
DB01765	(5-oxo-5,6-dihydro-indolo[1,2-a]quinazolin-7-yl)-acetic acid	447682	32	118	23		
DB02491	4-[4-(1-amino-1-methylethyl)phenyl]-5-chloro-n-[4-(2-morpholin-4-ylethyl)phenyl]pyrimidin-2-amine	447622	24	118	23		
DB11759	pevonedistat	16720766	33	117	23		
DB01167	itraconazole	55283	32	117	23	J02AC02	antimycotics for systemic use

DB01167	itraconazole	55283	32	117	23	J02AC02	antimycotics for systemic use
DB06144	sertindole	60149	29	117	23	N05AE03	psycholeptics
DB08091	3-fluoro-5-morpholin-4-yl-n-[3-(2-pyridin-4-ylethyl)-1h-indol-5-yl]benzamide	5326868	30	116	23		
DB12998	pf-00217830	11697676	30	116	23		
DB12368	azd-3839	46202416	28	116	23		
DB07608	n-(5-{[(2s)-4-amino-2-(3-chlorophenyl)butanoyl]amino}-1h-indazol-3-yl)benzamide	24894158	37	115	23		
DB07226	n-[4-(2-chlorophenyl)-1,3-dioxo-1,2,3,6-tetrahydropyrrolo[3,4-c]carbazol-9-yl]formamide	24778473	34	115	23		
DB07982	2-{4-[4-({4-[2-methyl-1-(1-methylethyl)-1h-imidazol-5-yl]pyrimidin-2-yl}amino)phenyl]piperazin-1-yl}-2-oxoethanol	11270500	25	115	23		
DB00248	cabergoline	54746	34	114	23	G02CB03	other gynecologicals
DB00248	cabergoline	54746	34	114	23	N04BC06	anti-parkinson drugs
DB00762	irinotecan	60838	32	114	23	L01XX19	antineoplastic agents
DB09206	trimazosin	37264	29	114	23	C02CA03	antihypertensives
DB06967	6-ethyl-5-[9-(3-methoxypropyl)-9h-carbazol-2-yl]pyrimidine-2,4-diamine	6914626	27	114	23		
DB08237	2'-deoxy-n-(naphthalen-1-ylmethyl)guanosine 5'-(dihydrogen phosphate)	46937134	39	113	23		
DB00685	trovafloxacin	62959	34	113	23	J01MA13	antibacterials for systemic use

DB00685	trovafloxacin	62959	34	113	23	J01MA13	antibacterials for systemic use
DB06191	zosuquidar	153997	33	113	23		
DB12654	gsk-376501	24946920	33	113	23		
DB05487	custirsen	72941942	38	112	23		
DB12222	lurtotecan	60956	32	112	23		
DB12050	ly-3039478	71236992	29	112	23		
DB02030	alpha-ribazole-5'-phosphate	444941	35	111	23		
DB12120	avoralstat	86566678	34	110	23		
DB11691	naldemedine	54732242	41	109	23		
DB01030	topotecan	60700	33	109	23	L01XX17	antineoplastic agents
DB04345	lumichrome	5326566	29	109	23		
DB02310	3,5,6,8-tetramethyl-n-methyl phenanthrolinium	168131	27	106	23		
DB01969	trifluoroacetonyl coenzyme a	449575	36	97	23		
DB00358	mefloquine	4046	30	97	23	P01BC02	antiprotozoals
DB13000	pci-27483	71587837	38	96	23		
DB04911	oritavancin	16136912	42	95	23	J01XA05	antibacterials for systemic use
DB04644	4-{4-[3-(2,4-dichloro-benzoyl)-ureido]-2,3-dimethyl-phenoxy}-butyric acid	5326886	31	94	23		
DB07074	6-carbamimidoyl-4-(3-hydroxy-2-methyl-benzoylamino)-naphthalene-2-carboxylic acid methyl ester	6852141	33	93	23		
DB12541	sar-405838	53476877	36	92	23		
DB02540	(10s)-10-formyl-5,8,10-trideazafolic acid	444725	33	92	23		
DB04264	(10r)-10-formyl-5,8,10-trideazafolic acid	444729	33	92	23		
DB09047	finafloxacin	11567473	38	91	23		

DB03531	flavin-adenine dinucleotide-n5-isobutyl ketone	131704249	37	91	23		
DB00218	moxifloxacin	152946	37	89	23	J01MA14	antibacterials for systemic use
DB00218	moxifloxacin	152946	37	89	23	J01MA14	antibacterials for systemic use
DB00218	moxifloxacin	152946	37	89	23	S01AE07	ophthalmologicals
DB08899	enzalutamide	15951529	30	89	23	L02BB04	endocrine therapy
DB01820	2-{[4-(2-acetylamino-2-pentylcarbamoyl-ethyl)-naphthalen-1-yl]-oxalyl-amino}-benzoic acid	447392	34	88	23		
DB08087	4-[(7r,7as)-7-hydroxy-1,3-dioxotetrahydro-1h-pyrrolo[1,2-c]imidazol-2(3h)-yl]-1-naphthonitrile	9944398	34	88	23		
DB12461	glpg-0492	59317190	30	86	23		
DB00365	grepafloxacin	72474	36	84	23	J01MA11	antibacterials for systemic use
DB12455	omadacycline	54697325	41	82	23		
DB06813	pralatrexate	148121	32	136	22	L01BA05	antineoplastic agents
DB08878	aminopterin	169371	29	136	22		
DB11805	saracatinib	10302451	35	135	22		
DB12694	ce-326597	52949124	34	135	22		
DB11652	tucatinib	51039094	32	134	22		
DB12096	pf-05175157	52934180	35	133	22		
DB12874	quizartinib	24889392	35	132	22		
DB07249	n-(5-chloro-1,3-benzodioxol-4-yl)-6-methoxy-7-(3-piperidin-1-ylpropoxy)quinazolin-4-amine	5330197	33	132	22		
DB11995	avatrombopag	9852519	29	131	22		

DB12062	volasertib	10461508	28	131	22		
DB11653	bremelanotide	9941379	31	130	22		
DB11703	acalabrutinib	71226662	28	130	22		
DB11747	barasertib	11497983	41	128	22		
DB06897	3-[3-chloro-5-(5-{[(1s)-1-phenylethyl]amino}isoxazolo[5,4-c]pyridin-3-yl)phenyl]propan-1-ol	24941248	40	128	22		
DB07321	2,5-dichloro-n-[5-methoxy-7-(6-methoxypyridin-3-yl)-1,3-benzoxazol-2-yl]benzenesulfonamide	6102821	32	128	22		
DB06999	n-{3-[(5-chloro-1h-pyrrolo[2,3-b]pyridin-3-yl)carbonyl]-2,4-difluorophenyl}propane-1-sulfonamide	24180719	31	128	22		
DB11730	ribociclib	44631912	29	128	22		
DB07704	6-amino-4-[2-(4-methoxyphenyl)ethyl]-1,7-dihydro-8h-imidazo[4,5-g]quinazolin-8-one	5326930	36	127	22		
DB07300	2-(1h-imidazol-1-yl)-9-methoxy-8-(2-methoxyethoxy)benzo[c][2,7]naphthyridin-4-amine	44176356	28	127	22		
DB02667	factor iiim	6323227	40	126	22		
DB11679	fruquintinib	44480399	38	126	22		

DB07460	2-({5-chloro-2-[(2-methoxy-4-morpholin-4-ylphenyl)amino]pyrimidin-4-yl}amino)-n-methylbenzamide	9934347	31	126	22		
DB04107	[(1-{2[(4-carbamimidoyl-phenylamino)-methyl]-1-methyl-1h-benzoimidazol-5-yl}-cyclopropyl)-pyridin-2-yl-methyleneaminooxy]-acetic acid ethyl ester	9600423	29	126	22		
DB12186	srt-2104	25108829	27	126	22		
DB05038	anatibant	9831652	36	125	22		
DB13013	lff-571	42638236	36	125	22		
DB12264	atevirdine	60848	31	125	22		
DB12297	glpg-0187	53340771	29	125	22		
DB03179	sinapoyl coenzyme a	131704232	41	124	22		
DB12785	furaprevir	51037069	38	124	22		
DB09297	paritaprevir	68498031	35	124	22		
DB03797	3-aminomethyl-pyridinium-adenine-dinucleotide	131704258	33	124	22		
DB01763	7-thionicotinamide-adenine-dinucleotide phosphate	131704180	32	124	22		
DB03893	thionicotinamide-adenine-dinucleotide	131704262	32	124	22		
DB11718	encorafenib	50922675	30	124	22		

DB02388	cyclohexyl-{4-[5-(3,4-dichlorophenyl)-2-piperidin-4-yl-3-propyl-3h-imidazol-4-yl]-pyrimidin-2-yl}amine	447873	27	124	22		
DB08613	2,2,2-trifluoro-1-{5-[(3-phenyl-5,6-dihydroimidazo[1,2-a]pyrazin-7(8h)-yl)carbonyl]thiophen-2-yl}ethane-1,1-diol	24836810	27	124	22		
DB11591	bilastine	185460	25	124	22	R06AX29	antihistamines for systemic use
DB08042	n~4~-methyl-n~4~-(3-methyl-1h-indazol-6-yl)-n~2~-(3,4,5-trimethoxyphenyl)pyrimidine-2,4-diamine	24941243	35	123	22		
DB06896	1-(4-fluorophenyl)-n-[3-fluoro-4-(1h-pyrrolo[2,3-b]pyridin-4-yloxy)phenyl]-2-oxo-1,2-dihydropyridine-3-carboxamide	21081761	30	123	22		
DB07045	(2r,3r,4s,5r)-2-[6-amino-8-[(3,4-dichlorophenyl)methylamino]purin-9-yl]-5-(hydroxymethyl)oxolane-3,4-diol	25195347	29	123	22		
DB07837	[4-(5-naphthalen-2-yl-1h-pyrrolo[2,3-b]pyridin-3-yl)phenyl]acetic acid	11952989	28	123	22		
DB11798	tanzisertib	11597537	27	123	22		

DB07152	n-[4-(5-fluoro-6-methylpyridin-2-yl)-5-quinoxalin-6-yl-1h-imidazol-2-yl]acetamide	25138294	25	123	22		
DB08676	(20s)-19,20,22,23-tetrahydro-19-oxo-5h,21h-18,20-ethano-12,14-etheno-6,10-methenobenz[d]imidazo[4,3-l][1,6,9,13]oxatriazacyclonoadecosine-9-carbonitrile	131704313	32	122	22		
DB08450	n-1h-indazol-5-yl-2-(6-methylpyridin-2-yl)quinazolin-4-amine	9998128	31	122	22		
DB03084	cyclopropyl-{4-[5-(3,4-dichlorophenyl)-2-[(1-methyl)-piperidin]-4-yl-3-propyl-3h-imidazol-4-yl]-pyrimidin-2-yl}amine	447872	27	122	22		
DB11951	lemborexant	56944144	25	122	22		
DB00619	imatinib	5291	24	122	22	L01XE01	antineoplastic agents
DB03613	4-hydroxyphenacyl coenzyme a	446968	41	121	22		
DB00457	prazosin	4893	32	121	22	C02CA01	antihypertensives
DB03325	tyrosyladenylate	449029	31	121	22		
DB01523	clonitazene	62528	28	121	22		
DB11763	momelotinib	25062766	24	121	22		
DB07227	4-[(5-{[4-(3-chlorophenyl)-3-oxopiperazin-1-yl]methyl}-1h-imidazol-1-yl)methyl]benzonitrile	216454	23	121	22		

DB00699	nicergoline	34040	45	120	22	C04AE02	peripheral vasodilators
DB02063	cra_16847	131704187	38	120	22		
DB12375	oglemilast	11387409	35	120	22		
DB08006	n-anthracen-2-yl-5-methyl[1,2,4]triazolo[1,5-a]pyrimidin-7-amine	24882195	29	120	22		
DB11903	gw842166	10253143	29	120	22		
DB00398	sorafenib	216239	28	120	22	L01XE05	antineoplastic agents
DB03373	zk-806711	131704242	32	119	22		
DB07474	3-[5-(1h-imidazol-1-yl)-7-methyl-1h-benzimidazol-2-yl]-4-[(pyridin-2-ylmethyl)amino]pyridin-2(1h)-one	22021390	29	119	22		
DB05478	pradefovir mesylate	9604653	35	118	22		
DB02316	1-(5-carboxypentyl)-5-[(2,6-dichlorobenzyl)oxy]-1 h-indole-2-carboxylic acid	11113761	31	118	22		
DB02226	3,8-diamino-6-phenyl-5-[6-[1-[2-[(1,2,3,4-tetrahydro-9-acridinyl)amino]ethyl]-1h-1,2,3-triazol-4-yl]hexyl]-phenanthridinium	5289507	29	118	22		
DB03005	3,8-diamino-6-phenyl-5-[6-[1-[2-[(1,2,3,4-tetrahydro-9-acridinyl)amino]ethyl]-1h-1,2,3-triazol-5-yl]hexyl]-phenanthridinium	5289508	29	117	22		

DB12225	beclabuvir	56934415	39	116	22		
DB06791	lanreotide	71349	36	116	22	H01CB03	pituitary and hypothalamic hormones and analogues
DB08031	n-[(13-cyclohexyl-6,7-dihydroindolo[1,2-d][1,4]benzoxazepin-10-yl)carbonyl]-2-methyl-l-alanine	11987832	34	116	22		
DB08865	crizotinib	11626560	32	116	22	L01XE16	antineoplastic agents
DB12535	ac-430	46892373	32	116	22		
DB00549	zafirlukast	5717	32	115	22	R03DC01	drugs for obstructive airway diseases
DB12307	foretinib	42642645	29	115	22		
DB12501	abt-384	11670435	29	115	22		
DB03383	5-chloro-1h-indole-2-carboxylic acid [1-(4-fluorobenzyl)-2-(4-hydroxypiperidin-1yl)-2-oxoethyl]amide	444746	32	114	22		
DB06741	gavestinel	6450546	30	114	22		
DB12659	sj-733	89508529	29	114	22		
DB12904	zstk-474	11647372	24	114	22		
DB03853	reactive red 6 hapten	6400893	41	113	22		
DB07981	2-[1-(4-chlorobenzoyl)-5-methoxy-2-methyl-1h-indol-3-yl]-n-[(1r)-1-(hydroxymethyl)propyl]acetamide	16741227	33	113	22		

DB07984	2-[1-(4-chlorobenzoyl)-5-methoxy-2-methyl-1h-indol-3-yl]-n-[(1s)-1-(hydroxymethyl)propyl]acetamide	16741228	33	113	22		
DB12635	aleplasinin	10224267	33	113	22		
DB00328	indomethacin	3715	31	113	22		
DB13783	acemetacin	1981	31	113	22	M01AB11	antiinflammatory and antirheumatic products
DB11977	golvatinib	16118392	28	113	22		
DB13874	enasidenib	89683805	23	113	22		
DB12381	merestinib	44603533	35	112	22		
DB13374	vincamine	15376	29	112	22	C04AX07	peripheral vasodilators
DB02008	1-(2-fluorobenzyl)-3-butyl-8-(n-acetyl-4-aminobenzyl)-xanthine	447371	29	111	22		
DB03044	1-(5-tert-butyl-2-p-tolyl-2h-pyrazol-3-yl)-3-[4-(2-morpholin-4-yl-ethoxy)-naphthalen-1-yl]-urea	156422	31	110	22		
DB12203	tozadenant	11618368	26	110	22		
DB12367	selurampanel	45381907	37	109	22		
DB01232	saquinavir	441243	31	109	22	J05AE01	antivirals for systemic use
DB04175	mdl-29951	446916	31	109	22		
DB11900	oc-459	11462174	25	108	22		
DB12178	telinavir	382974	30	107	22		
DB01095	fluvastatin	1548972	33	105	22	C10AA04	lipid modifying agents
DB07444	6-(3-aminopropyl)-4,9-dimethylpyrrolo[3,4-c]carbazole-1,3(2h,6h)-dione	6540255	33	105	22		

DB00247	methysergide	6540428	31	105	22	N02CA04	analgesics
DB04608	9-hydroxy-4-phenyl-6h-pyrrolo[3,4-c]carbazole-1,3-dione	4369491	36	104	22		
DB11814	pf-03882845	46871935	33	104	22		
DB13036	ramatroban	123879	29	104	22		
DB02633	cibacron blue	172469	37	103	22		
DB12382	r-306465	10309899	32	103	22		
DB12295	evodenoson	11627443	35	102	22		
DB11725	latrepirdine	197033	26	102	22		
DB05528	mipomersen	131704297	33	101	22	C10AX11	lipid modifying agents
DB06094	apatorsen	131704298	33	101	22		
DB04452	aminoquinuride	71166	24	101	22		
DB06219	dalbavancin	23724878	41	97	22	J01XA04	antibacterials for systemic use
DB12643	nelivaptan	9895468	38	96	22		
DB09101	elvitegravir	5277135	39	94	22	J05AX11	antivirals for systemic use
DB11560	lesinurad	53465279	30	94	22	M04AB05	antigout preparations
DB04881	elacridar	119373	33	93	22		
DB11847	cadazolid	44242317	35	92	22		
DB02455	fluoresceinylthioureido	49866988	31	91	22		
DB03481	5,10-dimethylene tetrahydromethanopterin	46936694	36	89	22		
DB11753	rifamycin	6324616	39	87	22	J04AB03	antimycobacterials
DB11753	rifamycin	6324616	39	87	22	S01AA16	ophthalmologicals
DB11753	rifamycin	6324616	39	87	22	S02AA12	otologicals
DB01044	gatifloxacin	5379	34	86	22	J01MA16	antibacterials for systemic use
DB01044	gatifloxacin	5379	34	86	22	J01MA16	antibacterials for systemic use

DB01044	gatifloxacin	5379	34	86	22	S01AE06	ophthalmologicals
DB07212	n-(7-carbamimidoyl-naphthalen-1-yl)-3-hydroxy-2-methyl-benzamide	6852140	31	86	22		
DB07022	3-hydroxypropyl 3-[(7-carbamimidoyl-1-naphthyl)carbamoyl]benzenesulfonate	11304895	30	86	22		
DB02164	n-sulfo-flavin mononucleotide	5288230	34	85	22		
DB03247	flavin mononucleotide	643976	34	85	22		
DB00140	riboflavin	493570	28	85	22		
DB09183	dasabuvir	56640146	30	82	22	J05AX16	antivirals for systemic use
DB00615	rifabutin	6323490	40	81	22	J04AB04	antimycobacterials
DB05861	tasquinimod	54682876	29	78	22		
DB12427	orvepitant	9852175	29	73	22		
DB11952	duvelisib	50905713	30	135	21		
DB12556	mk-5108	24748204	29	134	21		
DB11891	cudc-907	54575456	32	132	21		
DB06695	dabigatran etexilate	6445226	29	131	21	B01AE07	antithrombotic agents
DB11865	brivanib alaninate	11154925	33	130	21		
DB00402	eszopiclone	969472	29	129	21	N05CF04	psycholeptics
DB01198	zopiclone	5735	29	129	21	N05CF01	psycholeptics
DB08931	riociguat	11304743	25	129	21	C02KX05	antihypertensives
DB03062	(1-methyl-1h-imidazol-2-yl)-(3-methyl-4-{3-[(pyridin-3-ylmethyl)-amino]-propoxy}-benzofuran-2-yl)-methanone	446386	34	128	21		
DB11864	preladenant	10117987	30	128	21		
DB11708	peficitinib	57928403	32	127	21		

DB12597	abl-001	72165228	34	126	21		
DB07698	3-(3-aminophenyl)-n-(3-chlorophenyl)pyrazolo[1,5-a]pyrimidin-5-amine	6419789	26	126	21		
DB08096	8-(2-chlorophenylamino)-2-(2,6-difluorophenylamino)-9-ethyl-9h-purine-1,7-diium	6852209	25	126	21		
DB11765	pf-04991532	46181428	31	125	21		
DB12206	n-6022	44623946	27	125	21		
DB00976	telithromycin	3002190	29	124	21	J01FA15	antibacterials for systemic use
DB08437	puromycin	439530	29	124	21		
DB12776	e-6005	24864553	29	123	21		
DB12180	apitolisib	25254071	25	123	21		
DB03923	feruloyl coenzyme a	44229079	38	122	21		
DB06684	vilazodone	6918314	36	122	21	N06AX24	psychoanaleptics
DB08519	n~4~-(3-methyl-1h-indazol-6-yl)-n~2~-(3,4,5-trimethoxyphenyl)pyrimidine-2,4-diamine	9822610	34	122	21		
DB00290	bleomycin	5360373	33	122	21	L01DC01	antineoplastic agents
DB08267	6-amino-4-(2-phenylethyl)-1,7-dihydro-8h-imidazo[4,5-g]quinazolin-8-one	5326928	33	122	21		
DB07092	(2s,3s)-3-amino-4-(3,3-difluoropyrrolidin-1-yl)-n,n-dimethyl-4-oxo-2-(trans-4-[1,2,4]triazolo[1,5-a]pyridin-6-ylcyclohexyl)butanamide	11554165	29	122	21		

DB08504	6-(4-{(1s,2s)-2-amino-1-[(dimethylamino)carbonyl]-3-[(3s)-3-fluoropyrrolidin-1-yl]-3-oxopropyl}phenyl)-1h-[1,2,4]triazolo[1,5-a]pyridin-4-ium	10320144	29	122	21		
DB02848	{4-[3-(6,7-diethoxy-quinazolin-4-ylamino)-phenyl]-thiazol-2-yl}-methanol	5740	28	122	21		
DB11923	decoglurant	71533696	28	122	21		
DB12892	mgb-bp-3	23730143	27	122	21		
DB07159	tamatinib	11213558	26	122	21		
DB04751	purvalanol a	456214	25	122	21		
DB05413	tifuvirtide	16130644	38	121	21		
DB12494	sgi-1776	24795070	29	121	21		
DB04849	cediranib	9933475	28	121	21	L01XE32	antineoplastic agents
DB07643	5-{[1-(2,3-dichlorobenzyl)piperidin-4-yl]methoxy}quinazoline-2,4-diamine	24978486	28	121	21		
DB12364	betrixaban	10275777	28	121	21		
DB08896	regorafenib	11167602	27	121	21	L01XE21	antineoplastic agents
DB07889	(2s)-1-(dimethylamino)-3-(4-{[4-(2-methylimidazo[1,2-a]pyridin-3-yl)-2-pyrimidinyl]amino}phenoxy)-2-propanol	447653	26	121	21		

DB07379	(2s)-2-({6-[(3-amino-5-chlorophenyl)amino]-9-isopropyl-9h-purin-2-yl}amino)-3-methyl-1-butanol	6914609	25	121	21		
DB08325	2-({6-[(3-chlorophenyl)amino]-9-isopropyl-9h-purin-2-yl}amino)ethanol	2856	25	121	21		
DB01652	4-hydroxybenzoyl-coa	168718	40	120	21		
DB08350	5-[3-(2-methoxyphenyl)-1h-pyrrolo[2,3-b]pyridin-5-yl]-n,n-dimethylpyridine-3-carboxamide	16750094	32	120	21		
DB12963	ccx-354	25016615	32	120	21		
DB00969	alosetron	2099	31	120	21	A03AE01	drugs for functional gastrointestinal disorders
DB05294	vandetanib	3081361	35	119	21	L01XE12	antineoplastic agents
DB00275	olmesartan	158781	32	119	21		
DB12997	brilacidin	25023695	31	119	21		
DB00872	conivaptan	151171	30	119	21	C03XA02	diuretics
DB00590	doxazosin	3157	29	119	21	C02CA04	antihypertensives
DB06589	pazopanib	10113978	29	119	21	L01XE11	antineoplastic agents
DB07248	7-pyridin-2-yl-n-(3,4,5-trimethoxyphenyl)-7h-pyrrolo[2,3-d]pyrimidin-2-amine	11957393	28	119	21		
DB07117	5-(4-phenoxyphenyl)-5-(4-pyrimidin-2-ylpiperazin-1-yl)pyrimidine-2,4,6(2h,3h)-trione	10072851	27	119	21		
DB11758	cenicriviroc	11285792	27	119	21		

DB12703	omipalisib	25167777	27	119	21		
DB12570	cc-223	58298316	26	119	21		
DB08513	[4-({5-(aminocarbonyl)-4-[(3-methylphenyl)amino]pyrimidin-2-yl}amino)phenyl]acetic acid	24871491	25	119	21		
DB01369	quinupristin	5388937	34	118	21		
DB11886	infigratinib	53235510	31	118	21		
DB12725	td-8954	11961293	31	118	21		
DB11878	filibuvir	54708673	29	118	21		
DB12432	cc-401	10430360	33	117	21		
DB08789	2-amino-4-(2,4-dichlorophenyl)-n-ethylthieno[2,3-d]pyrimidine-6-carboxamide	44129601	28	117	21		
DB07750	(2r)-1-[4-({4-[(2,5-dichlorophenyl)amino]-2-pyrimidinyl}amino)phenoxy]-3-(dimethylamino)-2-propanol	445952	26	117	21		
DB07755	(2s)-1-[4-({4-[(2,5-dichlorophenyl)amino]-2-pyrimidinyl}amino)phenoxy]-3-(dimethylamino)-2-propanol	445953	26	117	21		
DB04011	2'-(4-dimethylaminophenyl)-5-(4-methyl-1-piperazinyl)-2,5'-bi-benzimidazole	448202	25	117	21		
DB04067	4-hydroxybenzyl coenzyme a	446969	37	116	21		
DB12044	mk-0893	11570626	35	116	21		

DB08683	rel-(9r,12s)-9,10,11,12-tetrahydro-9,12-epoxy-1h-diindolo[1,2,3-fg:3',2',1'-kl]pyrrolo[3,4-i][1,6]benzodiazocine-1,3(2h)-dione	447446	34	116	21		
DB12673	atx-914	119026214	32	116	21		
DB13125	lusutrombopag	49843517	32	116	21		
DB08774	1-[(2s)-4-(5-phenyl-1h-pyrazolo[3,4-b]pyridin-4-yl)morpholin-2-yl]methanamine	44129608	31	116	21		
DB12394	eleclazine	71183216	30	116	21		
DB01349	tasosartan	60919	28	116	21	C09CA05	agents acting on the renin-angiotensin system
DB08445	(3r,4s)-1-[6-(6-methoxypyridin-3-yl)pyrimidin-4-yl]-4-(2,4,5-trifluorophenyl)pyrrolidin-3-amine	24180585	27	116	21		
DB00206	reserpine	5770	39	115	21	C02AA02	antihypertensives
DB00206	reserpine	5770	39	115	21	C02AA02	antihypertensives
DB01089	deserpidine	8550	38	115	21	C02AA05	antihypertensives
DB01180	rescinnamine	32681	36	115	21	C02AA01	antihypertensives
DB08564	(2e)-n-{4-[(3-bromophenyl)amino]quinazolin-6-yl}-4-(dimethylamino)but-2-enamide	5328969	32	115	21		

DB11835	indium in-111 pentetreotide	131704321	31	115	21		
DB08816	ticagrelor	9871419	30	115	21	B01AC24	antithrombotic agents
DB01162	terazosin	5401	29	115	21	G04CA03	urologicals
DB08197	(5e,7s)-2-amino-7-(4-fluoro-2-pyridin-3-ylphenyl)-4-methyl-7,8-dihydroquinazolin-5(6h)-one oxime	46937132	25	115	21		
DB12064	bms-777607	24794418	25	115	21		
DB03678	(6,7-difluoro-quinazolin-4-yl)-(1-methyl-2,2-diphenyl-ethyl)-amine	5496989	24	115	21		
DB07280	5-[4-(dimethylamino)phenyl]-6-[(6-morpholin-4-ylpyridin-3-yl)ethynyl]pyrimidin-4-amine	11987868	24	115	21		
DB09285	morniflumate	72106	29	114	21	M01AX22	antiinflammatory and antirheumatic products
DB12746	akn-028	44177328	26	114	21		
DB01836	[4-(6-chloro-naphthalene-2-sulfonyl)-piperazin-1-yl]-(3,4,5,6-tetrahydro-2h-[1,4']bipyridinyl-4-yl)-methanone	446399	29	113	21		
DB07193	(2r,3r)-7-(methylsulfonyl)-3-(2,4,5-trifluorophenyl)-1,2,3,4-tetrahydropyrido[1,2-a]benzimidazol-2-amine	11710963	28	113	21		
DB13164	olmutinib	54758501	28	113	21		

DB07642	5-{[1-(2-fluorobenzyl)piperidin-4-yl]methoxy}quinazoline-2,4-diamine	23729147	26	113	21		
DB07063	{3-[(4,5,7-trifluoro-1,3-benzothiazol-2-yl)methyl]-1h-indol-1-yl}acetic acid	157839	24	113	21		
DB13141	ambroxol acefyllinate	176595	40	112	21		
DB06266	lonidamine	39562	31	112	21	L01XX07	antineoplastic agents
DB05713	ly-517717	9939865	29	112	21		
DB11773	bms-903452	53477157	23	112	21		
DB03311	3-(3,5-dibromo-4-hydroxy-benzoyl)-2-ethyl-benzofuran-6-sulfonic acid [4-(thiazol-2-ylsulfamoyl)-phenyl]-amide	448662	42	111	21		
DB08349	n-cyclopropyl-3-{[1-(2,4-difluorophenyl)-7-methyl-6-oxo-6,7-dihydro-1h-pyrazolo[3,4-b]pyridin-4-yl]amino}-4-methylbenzamide	42647297	32	111	21		
DB12198	fosdagrocorat	24872952	31	111	21		
DB03507	6-[3-(4-morpholinyl)propyl]-2-(3-nitrophenyl)-5-thioxo-5,6,-dihydro-7h-thienol[2',3':4,5]pyrrolo[1,2-c]imidazol-7-one	4470566	30	111	21		

DB08424	[5-amino-1-(4-fluorophenyl)-1h-pyrazol-4-yl](3-{[(2r)-2,3-dihydroxypropyl]oxy}phenyl) methanone	5327067	30	111	21		
DB07993	n~3~-[3-(1h-indol-6-yl)benzyl]pyridine-2,3-diamine	11507920	27	111	21		
DB07405	1-(6-cyano-3-pyridylcarbonyl)-5',8'-difluorospiro[piperidine-4,2'(1'h)-quinazoline]-4'-amine	9886249	26	111	21		
DB07054	(2r)-1-(dimethylamino)-3-{4-[(6-{[2-fluoro-5-(trifluoromethyl)phenyl]amino}pyrimidin-4-yl)amino]phenoxy}propan-2-ol	448994	25	111	21		
DB12866	pradigastat	53387035	21	111	21		
DB06448	lonafarnib	148195	36	110	21		
DB01772	3-[3-(2,3-dihydroxy-propylamino)-phenyl]-4-(5-fluoro-1-methyl-1h-indol-3-yl)-pyrrole-2,5-dione	448238	30	110	21		
DB12124	namitecan	10950142	30	110	21		
DB08164	(3r,4r)-1-{6-[3-(methylsulfonyl)phenyl]pyrimidin-4-yl}-4-(2,4,5-trifluorophenyl)piperidin-3-amine	16058650	25	110	21		
DB11896	gedatolisib	44516953	24	110	21		

DB07062	n-{3-[4-hydroxy-1-(3-methylbutyl)-2-oxo-1,2-dihydropyrrolo[1,2-b]pyridazin-3-yl]-1,1-dioxido-2h-1,2,4-benzothiadiazin-7-yl}methanesulfonamide	46937047	34	109	21		
DB07616	4-{[4-(4-fluoro-3-methylphenyl)-1,3-thiazol-2-yl]amino}-2-hydroxybenzoic acid	695906	33	109	21		
DB11822	tmc-647055	44556044	33	109	21		
DB07241	7-carboxy-5-hydroxy-12,13-dihydro-6h-indolo[2,3-a]pyrrolo[3,4-c]carbazole	46937063	30	109	21		
DB00932	tipranavir	54682461	24	109	21	J05AE09	antivirals for systemic use
DB07622	1-(3-(2,4-dimethylthiazol-5-yl)-4-oxo-2,4-dihydroindeno[1,2-c]pyrazol-5-yl)-3-(4-methylpiperazin-1-yl)urea	5288018	41	108	21		
DB01392	yohimbine	8969	32	108	21	G04BE04	urologicals
DB07247	[2'-hydroxy-3'-(1h-pyrrolo[3,2-c]pyridin-2-yl)-biphenyl-3-ylmethyl]-urea	15942655	28	108	21		
DB01611	hydroxychloroquine	3652	24	108	21	P01BA02	antiprotozoals
DB12459	belotecan	6456014	30	107	21		
DB05482	7-ethyl-10-hydroxycamptothecin	104842	29	107	21		
DB05806	cositecan	148202	28	107	21		

DB08099	6-ethyl-5-[(2s)-1-(3-methoxypropyl)-2-phenyl-1,2,3,4-tetrahydroquinolin-7-yl]pyrimidine-2,4-diamine	11963546	24	107	21		
DB08317	5-methyl-6-phenylquinazoline-2,4-diamine	25271556	25	106	21		
DB05009	apadenoson	9805430	33	105	21		
DB01967	n-{3-[(7ar,12as,12bs)-7-oxo-1,3,4,6,7,7a,12a,12b-octahydroindolo[2,3-a]quinolizin-12(2h)-yl]propyl}propane-2-sulfonamide	11987780	30	105	21		
DB12041	mapracorat	24795088	29	105	21		
DB07093	{3-[(5-chloro-1,3-benzothiazol-2-yl)methyl]-2,4-dioxo-3,4-dihydropyrimidin-1(2h)-yl}acetic acid	10450624	27	105	21		
DB11782	birinapant	49836020	27	105	21		
DB00224	indinavir	5362440	34	104	21	J05AE02	antivirals for systemic use
DB00353	methylergometrine	8226	30	104	21	G02AB01	other gynecologicals
DB00353	methylergometrine	8226	30	104	21	G02AB01	other gynecologicals
DB01253	ergometrine	443884	30	104	21	G02AB03	other gynecologicals
DB01253	ergometrine	443884	30	104	21	G02AB03	other gynecologicals
DB08860	pitavastatin	5282452	29	103	21	C10AA08	lipid modifying agents
DB04934	rifalazil	6540558	39	102	21		
DB00467	enoxacin	3229	30	102	21	J01MA04	antibacterials for systemic use
DB02654	6-hydroxy-fad	4369241	34	101	21		

DB06856	6-fluoro-2-[2-hydroxy-3-(2-methyl-cyclohexyloxy)-phenyl]-1h-indole-5-carboxamidine	445849	30	101	21		
DB12405	triciribine	65399	27	100	21		
DB07756	1-[3-({[(4-amino-5-fluoro-2-methylquinolin-3-yl)methyl]thio}methyl)phenyl]-2,2,2-trifluoroethane-1,1-diol	1723	25	100	21		
DB03182	alpha-fluoro-carboxymethyldethia coenzyme a complex	444883	35	98	21		
DB04158	6-(adenosine tetraphosphate-methyl)-7,8-dihydropterin	6323200	33	97	21		
DB07395	4-[3-(2-chloro-4,5-difluoro-benzoyl)ureido]-3-trifluoromethoxybenzoic acid	16070039	27	96	21		
DB07396	1-{2-[3-(2-chloro-4,5-difluoro-benzoyl)-ureido]-4-fluoro-phenyl}-piperidine-4-carboxylic acid	12148754	24	96	21		
DB06441	cangrelor	9854012	32	94	21	B01AC25	antithrombotic agents
DB07061	1-(cyclohexylamino)-3-(6-methyl-3,4-dihydro-1h-carbazol-9(2h)-yl)propan-2-ol	961704	28	94	21		
DB08010	(3z)-1-[(6-fluoro-4h-1,3-benzodioxin-8-yl)methyl]-4-[(e)-2-phenylethenyl]-1h-indole-2,3-dione 3-oxime	45268539	33	93	21		

DB08015	(3z)-1-[(6-fluoro-4h-1,3-benzodioxin-8-yl)methyl]-4-phenyl-1h-indole-2,3-dione 3-oxime	45270264	33	93	21		
DB06402	telavancin	3081362	45	91	21	J01XA03	antibacterials for systemic use
DB12309	gi-181771x	51353551	32	91	21		
DB05109	trabectedin	108150	36	90	21	L01CX01	antineoplastic agents
DB03541	10-propargyl-5,8-dideazafolic acid	443388	35	90	21		
DB00524	metolazone	4170	28	88	21	C03BA08	diuretics
DB06920	eribaxaban	11634458	25	88	21		
DB12414	usistapide	56842069	24	88	21		
DB06212	tolvaptan	443894	34	87	21	C03XA01	diuretics
DB11774	pazufloxacin	65957	34	87	21	J01MA18	antibacterials for systemic use
DB01405	temafloxacin	60021	32	87	21	J01MA05	antibacterials for systemic use
DB11491	sarafloxacin	56208	31	87	21		
DB07238	nesbuvir	11561383	37	85	21		
DB11892	prulifloxacin	65947	31	84	21	J01MA17	antibacterials for systemic use
DB09003	clocapramine	2793	27	84	21		
DB12724	azd-7295	57339445	25	84	21		
DB11761	tenapanor	71587953	28	83	21		
DB08278	1-(2-cyclopropylethyl)-3-(1,1-dioxo-2h-1,2,4-benzothiadiazin-3-yl)-6-fluoro-4-hydroxy-2(1h)-quinolinone	5273394	26	83	21		
DB04786	suramin	5361	32	82	21		
DB06207	silodosin	5312125	32	82	21	G04CA04	urologicals

DB03605	(2s)-2-[(2,4-dichloro-benzoyl)-(3-trifluoromethyl-benzyl)-amino]-3-phenyl-propionic acid	447368	25	82	21		
DB02636	9-hydroxy-8-methoxy-6-nitro-phenanthrol[3,4-d][1,3]dioxole-5-carboxylic acid	1941	38	80	21		
DB00978	lomefloxacin	3948	32	80	21	J01MA07	antibacterials for systemic use
DB00978	lomefloxacin	3948	32	80	21	S01AE04	ophthalmologicals
DB04576	fleroxacin	3357	30	80	21	J01MA08	antibacterials for systemic use
DB04576	fleroxacin	3357	30	80	21	J01MA08	antibacterials for systemic use
DB01208	sparfloxacin	60464	32	79	21	J01MA09	antibacterials for systemic use
DB01734	3-(oxalyl-amino)-naphthalene-2-carboxylic acid	1628	30	79	21		
DB07323	2-[({2-[(1z)-3-(dimethylamino)prop-1-enyl]-4-fluorophenyl}sulfonyl)amino]-5,6,7,8-tetrahydronaphthalene-1-carboxylic acid	6102686	30	79	21		
DB04842	fluspirilene	3396	25	79	21	N05AG01	psycholeptics
DB13103	gpx-150	10392558	41	77	21		
