# Supplementary material for: Computationally prioritized drugs inhibit SARS-CoV-2 infection and syncytia formation
Source: Brief Bioinform. 2021 Dec 27;23(1):bbab507. doi: 10.1093/bib/bbab507 (PMC8769897; doi:10.1093/bib/bbab507)
Supplement: Table_S5_bbab507 [file table_s5_bbab507.pdf]

Table S5: Targets of 7-hydroxystaurosporine and bafetinib

	Target	Validated	IC50	Dose tested	Signaling pathway
**7-hydroxystaurosporine**	PDK1	experimentally	5 nM	-	Pi3K-AKT/ HIF-1
	CHK1	experimentally	-	1 micromolar	p53
	PKCa	experimentally	-	1 micromolar	WNT/ MAPK/ ErbB/ Ras/ Rap1/ Calcium/ HIF-1/ Phosphatidylinositol/ Sphingolipid / Phospholipase D/ mTOR / PI3K-Akt
	AMPK	experimentally	-	1 micromolar	AMPK/ FoxO/ mTOR/ Pi3K-AKT
	PHK	experimentally	-	1 micromolar	Calcium/ Insulin/ Glucagon
	LCK	experimentally	-	1 micromolar	NFkB/ T cell receptor
	CDK2	experimentally	-	1 micromolar	Pi3K-AKT/ FoxO/ p53
	PBK	experimentally	-	1 micromolar	Pi3K-AKT
	ROCK2	experimentally	-	1 micromolar	cAMP/ WNT/ cGMP-PKG/ Chemokine/ Sphingolipid
	S6k1	experimentally	-	1 micromolar	ERB/ AMPK/ HIF-1/ mTOR/ TGF-beta/ PI3K-Akt/ Apelin/ Insulin
	GSK3B	experimentally	-	1 micromolar	ERB/ T cell receptor/ mTOR/ EGFR/ Chemokine/ Hedgehog/ Hippo
	MAPKAP-k1	experimentally	-	1 micromolar	MAPK
	SGK1	experimentally	-	1 micromolar	FoxO/ Pi3K-AKT/ mTOR

	Target	Posssible Stages of infection Involvement	Gene Ontology/ KEGG pathway	Reference
**7-hydroxystaurosporine**	PDK1	viral genome replication, host cell signaling and Regulation	G.O.0019079; map04151; map04066	G.O.0019080, https://doi.org/10.15252/msb.202110239
	CHK1	viral genome replication	G.O.0019079; map04115	doi: 10.1128/mBio.03423-19
	PKCa	viral genome replication, endosomal maturation, host cell signaling and regulation	G.O.0019079;map04066;map04310;map04010;map04012;map00905;map04015;map04020;map00563;map04071;map04072;map04150;map04151	G.O.0019080, https://doi.org/10.15252/msb.202110239
	AMPK	host cell signaling and Regulation	map04151; map04150; map04152; map04068	https://doi.org/10.15252/msb.202110239
	PHK	Endosomal maturation	map04020; map04910; map04922	https://doi.org/10.15252/msb.202110239
	LCK	Immune response to the virus	map04064; map04660	https://doi.org/10.15252/msb.202110239, Reactome
	CDK2	host cell signaling and Regulation	map04151; map04115; map04068	10.1371/journal.pone.0057340
	PBK	host cell signaling and Regulation, viral genome replication	map04151	https://doi.org/10.15252/msb.202110239
	ROCK2	viral genome replication, Immune response to the virus	G.O.0019079; map04151; map04310; map04024; map04022; map04062	G.O.0019080, https://doi.org/10.15252/msb.202110239
	S6k1	Immune response to the virus, cell signaling and regulation	map04151;map04066;map04012;map04150;map04152;map04910;map04350;map04371	https://doi.org/10.15252/msb.202110239
	GSK3B	Immune response to the virus, cell signaling and regulation, viral gene expression	G.O.0019080; map04150; map04660; map04062; map04657; map04340; map04391	https://doi.org/10.15252/msb.202110239
	MAPKAP-k1	-	map04010	https://doi.org/10.15252/msb.202110239
	SGK1	cell signaling and regulation	map04151; map04150; map04068	https://doi.org/10.15252/msb.202110239

	Target	Validated	Signaling pathway	IC50	Dose tested
**Bafetinib**	ABL1	experimentally	ErB/ Ras	-	10 nM
	LYN	experimentally	Chemokine/ NFkB/ B cell receptor/ Fc epsilon RI	-	10 nM
	ABCG2	virtual screening and docking	NFkB/ Pi3K-AKT	-	-

	**Target**	**Posssible Stages of infection Involvement**	**Gene Ontology/ KEGG pathway**	**Reference**
**Bafetinib**	ABL1	*viral entry*	map04012; map00905	10.3389/fmicb.2017.01129
	LYN	Immune response to the virus	map04064; map04062; map04662; map04664	https://doi.org/10.15252/msb.202110239
	ABCG2	Immune response to the virus; viral genome replication	G.O.0019079; map04151; map04064;	10.1016/j.etap.2017.12.011; doi: 10.3892/or.2019.6968

Stages of infection	Pathways involved
**Endosomal maturation**	TLR signaling, Pi3P signaling pathway, Calcium signaling pathway
**Replication and Translation**	DNA replication, RNA transport, RNA degradation, Protein processing
**Cell signaling and Regulation**	mTOR signaling, PI3K-AKT signalling, AMPK signaling, autograp
**Immune response**	TLR signaling, chemokine signaling, NFkB ssignaling, RLR signaling, TCR signaling, BCR signaling, HIF-1 signaling
**Virus exit**	Cathepin B/L
